# E3‐Ubiquitin Ligase SgATL31 Promotes Anthracnose Resistance in *Stylosanthes* by Modulating ROS Burst and Antioxidant Defence: A Proteomic and Functional Study

**DOI:** 10.1111/mpp.70122

**Published:** 2025-07-07

**Authors:** Liyun Yang, Yunpiao Long, Mengze Gao, Shizi Zhang, Jing Gao, Lijuan Luo, Lingyan Jiang

**Affiliations:** ^1^ School of Breeding and Multiplication (Sanya Institute of Breeding and Multiplication), School of Tropical Agriculture and Forestry Hainan University Sanya Hainan China

**Keywords:** *Colletotrichum gloeosporioides*, evaluation of disease‐resistant germplasm resources, phosphoproteomics, plasma membrane enriched proteomics, SgATL31, *Stylosanthes*

## Abstract

*Stylosanthes* spp. (stylo) is an important leguminous forage cultivated in tropical areas. Anthracnose caused by *Colletotrichum gloeosporioides* is a destructive disease that limits the yield of stylo. Therefore, improving the resistance of stylo is crucial to control stylo anthracnose. In this study, the resistance evaluation of 40 Chinese stylo accessions was performed, including the main cultivar *Stylosanthes guianensis* ‘Reyan No. 2’ (RY2) as a susceptible control. Twelve stylo accessions were rated as highly resistant, with 2001–84 showing the strongest resistance. Compared to RY2, 2001‐84 exhibited significantly milder disease symptoms, slower fungal colonisation, and higher pathogen‐induced antioxidant enzyme activities. Integrated phosphoproteomics and plasma membrane (PM) enriched proteomics of both RY2 and 2001‐84 revealed that pathogen‐responsive proteins were predominantly associated with kinase signalling, transport processes, and oxidoreductase activity. A PM‐localised E3 ubiquitin ligase, SgATL31, was identified as increasing in response to pathogen in both proteomic analyses. Functional characterisation demonstrated that *SgATL31* overexpression in *Arabidopsis* enhanced resistance to *C. gloeosporioides*, promoted chitin‐induced reactive oxygen species (ROS) production in both *Arabidopsis* and stylo protoplasts, and increased antioxidant enzyme activities following pathogen infection. Furthermore, the expression levels of *SgATL31* were induced by pathogen infection in all 40 stylo accessions and accumulated to higher levels in resistant accessions. Overall, our findings not only identify 2001‐84 as a valuable genetic resource for anthracnose resistance but also establish SgATL31 as a regulator of plant immunity against anthracnose, potentially through modulation of ROS and antioxidant pathways, providing important insights for improving disease resistance in stylo.

## Graphical abstract

Integrated proteomics revealed anthracnose resistance factors in *Stylosanthes*, identifying the E3 ligase SgATL31 as a positive regulator through ROS and antioxidant pathway modulation.

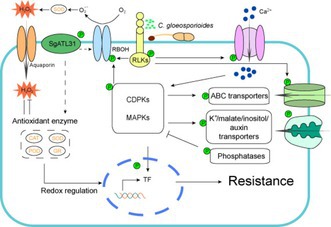

## 1 Introduction

*Stylosanthes* (stylo), a prominent leguminous forage crop widely cultivated in tropical and subtropical regions (Calles and Schultze‐Kraft 2016; Clericuzio et al. 2017), serves dual agricultural roles as both high‐quality forage and effective cover crops, offering significant economic and ecological value (Schultze‐Kraft et al. 2023). These species exhibit remarkable adaptability to challenging soil conditions, demonstrating exceptional tolerance to phosphorus deficiency, drought stress, and aluminium/manganese toxicity (Han et al. 2022; Luo et al. 2020; Sun et al. 2013; Wu, Zhao, et al. 2023; Zou et al. 2022). Despite these advantages, stylo production faces severe constraints from anthracnose caused by *Colletotrichum gloeosporioides*, a devastating disease that significantly reduces yield and limits utilisation (Weeds et al. 2003). Current anthracnose management primarily relies on physical and chemical approaches (Schultze‐Kraft et al. 2023), yet these methods pose environmental concerns by potentially altering field ecosystems and soil composition (Linhart et al. 2019; Stephens and Kaminski 2019). The development of resistant cultivars represents a more sustainable and cost‐effective solution for disease control (Pathania et al. 2021). While previous screening of 358 stylo accessions identified several highly resistant accessions (Cameron et al. 1978), the limited durability of resistance, typically lasting about 10 years (Chen et al. 2014), underscores the ongoing need for continuous identification of novel resistant varieties, elucidation of resistance mechanisms, and characterisation of key resistance genes. Addressing these challenges remains critical for the sustainable development of the stylo industry.

Several studies have been performed on the resistance of stylo to anthracnose. Improved stylo resistance to *C. gloeosporioides* has been observed by increasing the activity of antioxidant enzymes and the expression levels of stress‐responsive genes, such as pathogenesis‐related genes (*PR1* and *PR5*), chitinase (*Cht*), and phenylalanine ammonia‐lyase (*PAL*) (Wang et al. 2017). Transcriptomics combined with metabolomics revealed that stylo responded to *C. gloeosporioides* infection by up‐regulating genes and metabolites in phenylpropanoid and flavonoid synthesis pathways (Jiang et al. 2021). In addition, the exogenous application of metabolites of phloretin and pterostilbene alleviated anthracnose by improving the antioxidant capacity and reducing the damage of chloroplasts (Zhang et al. 2024). Due to the unassembled genomes and genetic transformation limitations of stylo, the interaction system between *Arabidopsis thaliana* and *C. gloeosporioides* that could co‐infect both stylo and *Arabidopsis* with comparable virulence has been developed to explore anthracnose resistance mechanisms (Gao et al. 2021). However, the genes that contribute to the resistance and their related regulatory mechanisms need to be further explored.

As a barrier between the external environment and the intracellular cytoplasm, the plasma membrane (PM) serves as a crucial line of defence for plants against the invasion of pathogens (Yu et al. 2017). Pattern recognition receptors (PRRs) on the PM recognise microbial/pathogen‐associated molecular patterns (MAMPs/PAMPs) from pathogens and damage‐associated molecular patterns (DAMPs) from self‐damage, which triggers pattern‐triggered immunity (PTI). The immune responses include the induction of defence genes, reactive oxygen species (ROS) burst, calcium influx, and the activation of multiple protein kinases such as mitogen‐activated protein kinase (MAPKs) and calcium‐dependent protein kinases (CDPKs) (Jones et al. 2024). Various protein kinase pathways often coordinate the external and internal cues through protein phosphorylation to regulate the key cellular responses (Zhang et al. 2024). Therefore, both PM proteomics and phosphoproteomics could be effective ways to investigate changes in the plants in response to pathogen infection, and to identify potential proteins regulating the resistance. In rice, PM proteomics revealed stage‐specific responses to *Magnaporthe oryzae*: early jasmonic acid signalling activation followed by later cytokinin pathway induction and nutrient efflux (Cao et al. 2016). The integration of PM proteome, cytosolic proteome, and phosphoproteome of rice leaves identified MSP1 effector‐triggered redox metabolism and phosphorylation cascades involving RBOHD, MEKK1 and MPK3/6 (Gupta et al. 2019). Phosphoproteomics has also delineated resistance mechanisms, linking Rac GTPase and H_2_O_2_ signalling to cultivar‐specific immunity in rice (Li et al. 2015), and revealing OSCA1.3‐BIK1 interactions controlling stomatal immunity in *Arabidopsis* (Thor et al. 2020). These cases highlight how spatial (PM‐focused) and post‐translational (phosphorylation) proteomics can precisely map defence networks.

This study systematically evaluates anthracnose resistance across 40 stylo accessions using the most common cultivar in China, RY2, as a susceptible control. By integrating comparative proteomic analyses of resistant and susceptible genotypes, we aim to (1) identify resistance‐associated proteins, (2) determine defence response deficiencies in RY2 compared to more resistant accessions, and (3) provide potential genetic targets for breeding resistant cultivars. Our findings will advance the understanding of legume–fungal pathogen interactions and contribute to sustainable anthracnose management in forage crops.

## 2 Results

### 2.1 Resistance Evaluation of 40 Stylo Accessions Against *C. gloeosporioides*

**FIGURE 1:**
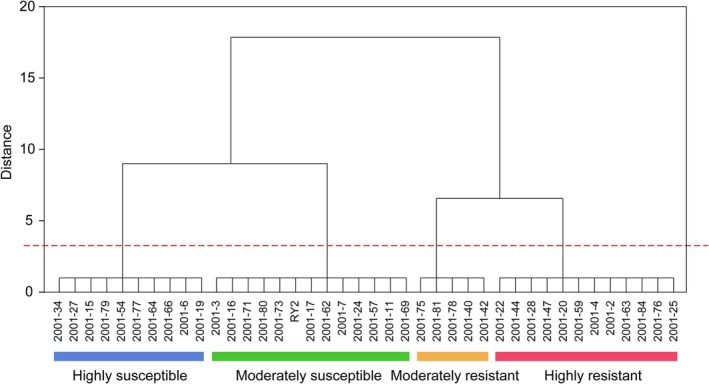
Cluster analysis of the disease resistance of 40 stylo accessions against *Colletotrichum gloeosporioides*. Red, yellow, green and blue indicate highly resistant, moderately resistant, moderately susceptible and highly susceptible, respectively. RY2 refers to *Stylosanthes guianensis* ‘Reyan No. 2’, the cultivar most widely grown in China.

The pathogen assay was performed on the 4‐week‐old plants of 40 stylo accessions by spray‐inoculation of spore suspension of *C. gloeosporioides* using RY2 as control. Five resistance indexes, namely leaf disease severity (LRAT), stem disease severity (SRAT), percentage of defoliation (DEFL), area of lesions per leaf (ALES) and dry weight index (DRWT), were measured and used to evaluate the resistance (Figure S1). Correlation analysis of resistance indexes showed that there were significant positive correlations among the five indexes (Figure S2). Principal component analysis (PCA) of resistance index showed that LRAT, SRAT, DEFL, ALES and DRWT were all important indexes reflecting the resistance of stylo, explaining 81.6% of the original data (Table S1). Comprehensive analysis of five resistance indexes showed that 12 (30%) accessions were rated as highly resistant (HR), 5 (12.5%) as moderately resistant (MR), 13 (32.5%) as moderately susceptible (MS), and 10 (25%) as highly susceptible (HS) (Figure 1). Discriminant analysis of the above results showed that 37 accessions (92.5%) were consistently classified by the comprehensive analysis, except for the 2001‐11, 2001‐24, and 2001‐78 (Table S2). Accession 2001‐84 was the most resistant with the lowest D value among the HR accessions (Table S3). Therefore, the 2001‐84 was chosen as the resistant accession, and the RY2 as the susceptible control for the following comparative studies to investigate the resistance mechanisms.

### 2.2 Comparison of Resistance Phenotypes of RY2 and 2001–84

**FIGURE 2:**
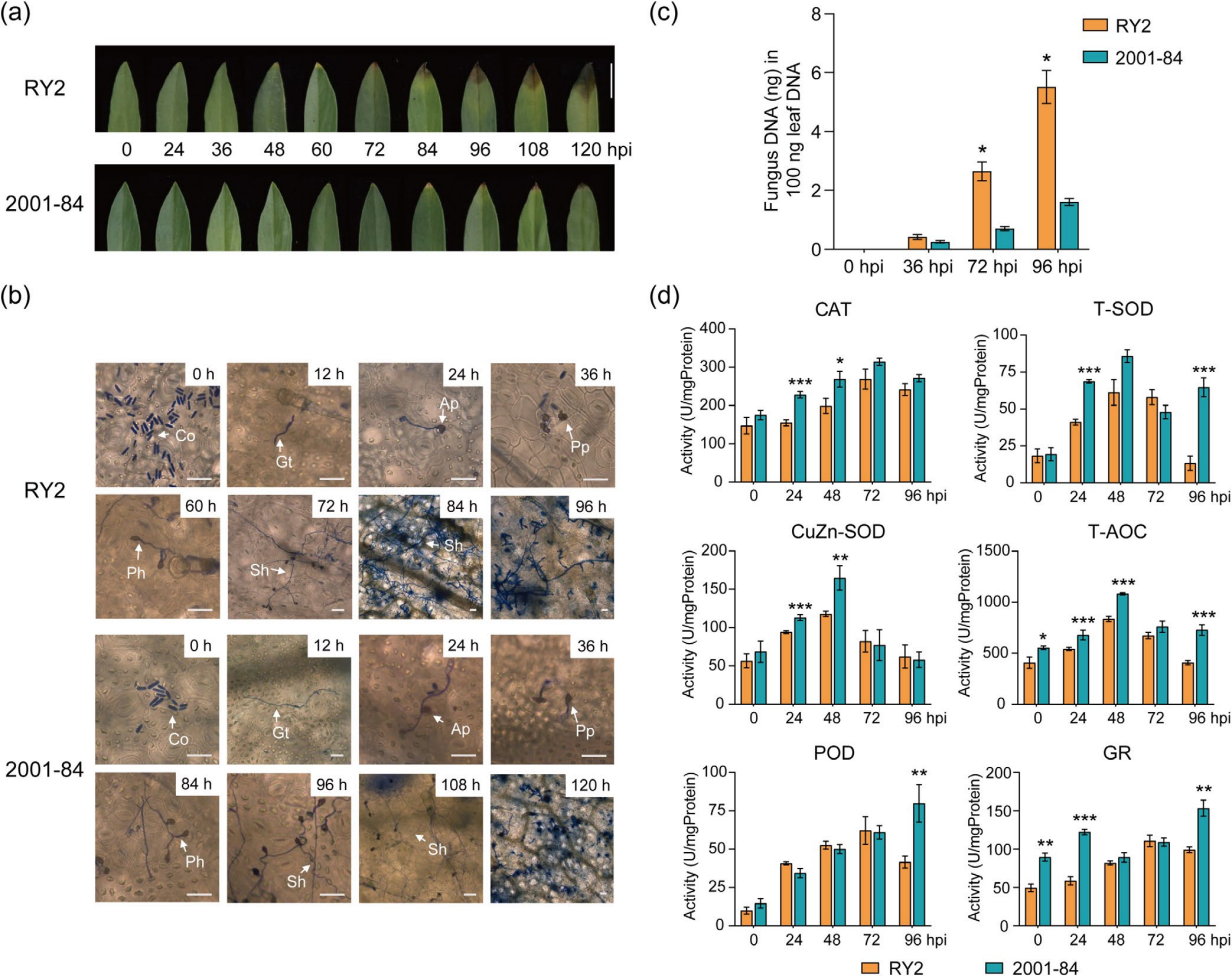
The resistance phenotypes of stylo RY2 and 2001‐84 after inoculation of *Colletotrichum gloeosporioides*. (a) Disease phenotypes of RY2 and 2001‐84 at 0, 24, 36, 48, 60, 72, 84, 96, 108, 120 h post‐inoculation (hpi), bar = 1 cm. (b) The development of *C. gloeosporioides* in RY2 and 2001‐84 after inoculation. Co, condia; Gt, germ tube; Ap, appressorium; Pp, penetration peg; Ph, primary hypha; Sh, secondary hypha. Bar = 40 μm. (c) The accumulation of *C. gloeosporioides* in RY2 and 2001‐84 at 36, 72, 96 hpi. Data are means ± SE pooled from three replicates (*n* = 3). (d) The determination of the antioxidant enzyme activities in RY2 and 2001‐84 after inoculation. CAT, catalse; T‐SOD, total superoxide dismutase; CuZn‐SOD, copper‐zinc superoxide dismutase; T‐AOC, total antioxidant capacity; POD, peroxidase; GR, glutathione reductase. Data are means ± SE pooled from three replicates (*n* = 4–8). Statistical significance is assessed in Student's *t* tests. Significant differences are observed between RY2 and 2001‐84 (*p < 0.05, **p < 0.01, ***p < 0.001).

To compare resistance and disease phenotypes, pathogen infection progression, and antioxidant enzyme activities, RY2 and 2001‐84 were inoculated with *C. gloeosporioides*. Leaf disease analysis revealed that RY2 exhibited yellowing at 48 h post‐inoculation (hpi) and dark brown necrotic lesions by 72 hpi, with lesions expanding thereafter. In contrast, 2001–84 showed delayed symptoms, with yellowing at 84 hpi and limited necrosis at 120 hpi (Figure 2a). Microscopic observation indicated that in RY2, *C. gloeosporioides* conidia germinated by 12 hpi, formed appressoria by 24 hpi, and produced penetration pegs by 36 hpi. Primary hyphae emerged at 60 hpi, followed by narrower secondary hyphae from 72 hpi, marking the shift from biotrophy to necrotrophy. By 84 hpi, secondary hyphae spread intercellularly, accompanied by new conidia. In 2001‐84, hyphal formation was delayed by 24 h (Figure 2b). Consistent with these findings, quantitative PCR (qPCR) confirmed higher *C. gloeosporioides* DNA levels in RY2 at 72 and 96 hpi (Figure 2c), indicating slower disease progression and limited pathogen infection in 2001‐84 compared to RY2.

Antioxidant enzyme activity plays a crucial role in plant resistance to pathogen infection, with stylo exhibiting increased enzyme activity following *C. gloeosporioides* inoculation (Wang et al. 2017). To compare antioxidant responses between RY2 and 2001‐84, we measured the activities of catalase (CAT), total superoxide dismutase (T‐SOD), copper‐zinc superoxide dismutase (CuZn‐SOD), total antioxidant capacity (T‐AOC), peoxidase (POD) and glutathione reductase (GR) at 0, 24, 48, 72, and 96 hpi (Figure 2d). Both RY2 and 2001‐84 showed similar single‐peak trends for CAT, T‐SOD, CuZn‐SOD and T‐AOC, but 2001‐84 exhibited higher overall activity. For POD and GR, RY2 displayed an initial rise followed by a decline, whereas 2001‐84 showed a steady increase from 0 to 96 hpi. These findings suggest that the enhanced antioxidant enzyme activity in 2001‐84 may contribute to its stronger resistance compared to RY2.

### 2.3 Phosphoproteomic Analysis of RY2 and 2001‐84 in Response to *C. gloeosporioides*

Protein phosphorylation is essential for immune responses in plants during pathogen invasion. To investigate the potential resistance mechanism of stylo against *C. gloeosporioides*, comparative phosphoproteomic analyses were performed on RY2 and 2001‐84. The transition from the biotrophic phase to the necrotrophic phase plays vital roles for hemibiotrophic pathogens to colonise the host and cause diseases. Therefore, 0 and 96 hpi were chosen as sampling time points, where 0 hpi was the control (CK) and 96 hpi was the transition time point in the resistant accession. A total of 14,018 phosphopeptides, 7339 non‐redundant phosphosites and 2507 phosphoproteins were identified. The peptides containing one, two or three phosphosites accounted for 96.91%, 3.06% or 0.03%, respectively (Figure S3a). The phosphosites of serine, threonine and tyrosine accounted for 70.42%, 22.8% and 6.78%, respectively (Figure S3b). PCA showed that the samples were significantly separated between CK and inoculation treatment, as well as different accessions, indicating that the data was reliable (Figure S3c). Genotypic comparisons between 2001‐84 and RY2 revealed 539 and 244 differentially phosphorylated proteins (DPPs) under CK conditions and at 96 hpi, respectively (Figure S3d, Tables S4 and S5). Temporal comparisons between 0 hpi (CK) and 96 hpi identified 512 and 485 DPPs in RY2 and 2001‐84, respectively (Figure S3d, Tables S6 and S7).

**FIGURE 3:**
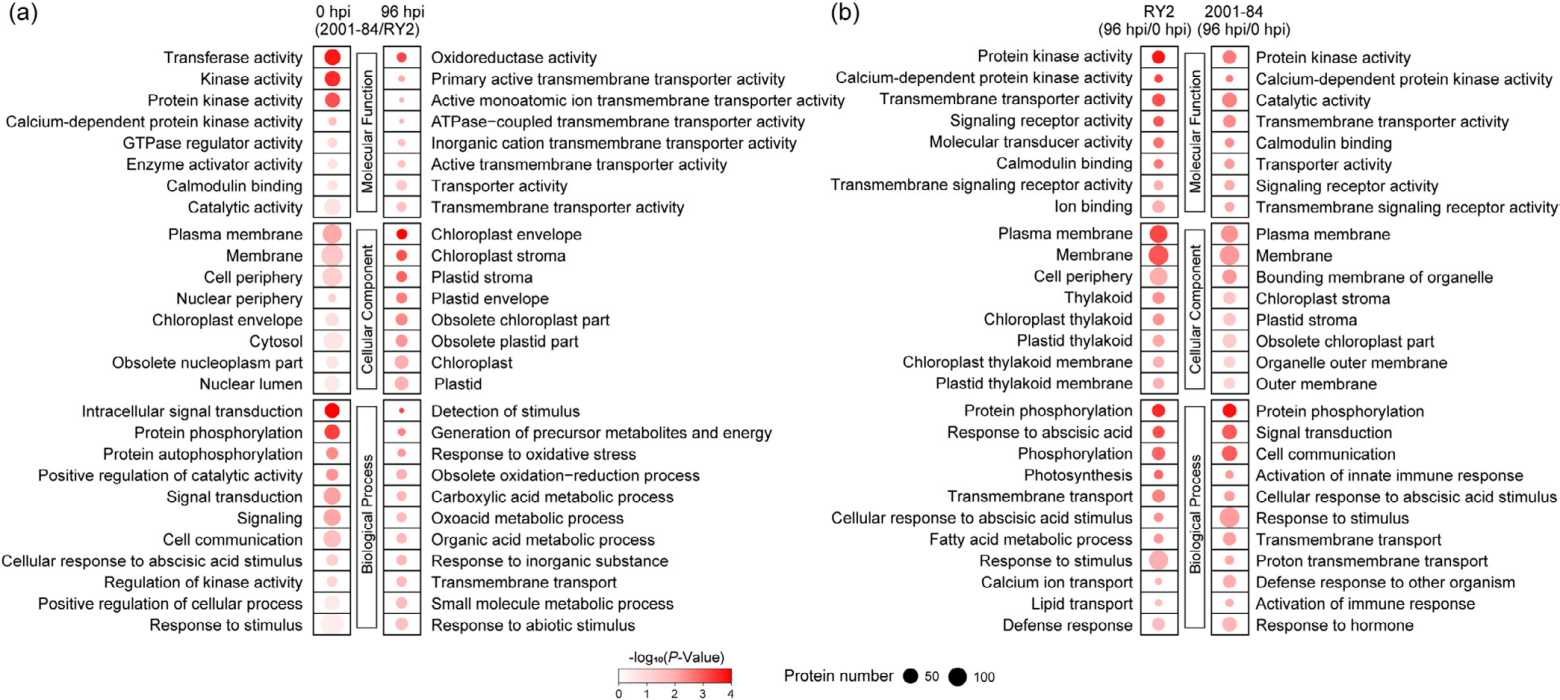
Gene Ontology (GO) enrichment analysis of differentially phosphorylated proteins (DPPs) in RY2 and 2001‐84. (a) Significantly enriched GO terms (*p* < 0.05) for DPPs identified between the resistant accession 2001‐84 and susceptible cultivar RY2 at 0 and 96 h post‐inoculation (hpi). (b) Enriched GO terms for DPPs between 96 hpi and 0 hpi within each genotype. GO terms are ranked vertically by statistical significance (−log_10_(*p*‐value)). Circle colour intensity represents the enrichment fold‐change (darker = higher enrichment), while circle size corresponds to the number of mapped proteins per term.

To investigate the function of DPPs, Go Ontology (GO) analysis was performed. Comparative analysis between 2001‐84 and RY2 at 0 hpi identified the most significant DPP enrichment in transferase activity (Molecular Function), plasma membrane (Cellular Component), and intracellular signal transduction (Biological Process). The enrichment pattern at 96 hpi shifted to oxidoreductase activity, chloroplast envelope, and detection of stimulus (Figure 3a, Tables S8 and S9). Subsequent temporal analysis (96 hpi vs. 0 hpi) revealed the most prominent enrichment in protein kinase activity, plasma membrane and protein phosphorylation in both genotypes (Figure 3b, Tables S10 and S11).

Given the central roles of receptor‐like kinases (RLKs), CDPKs and MAPKs in defence signalling, we conducted a comparative analysis of their temporal phosphorylation patterns between genotypes. The analysis identified a total of 21 RLKs, among which 5 RLKs were significantly up‐regulated in both genotypes. Notably, 6 RLKs exhibited exclusive up‐regulation in RY2, while 5 were specifically up‐regulated in 2001‐84. In contrast, 4 and 1 RLKs were uniquely suppressed in RY2 and 2001–84, respectively (Figure S4, Table S12). These findings indicate that pathogen‐induced RLK phosphorylation activates defence signalling in both genotypes. Intriguingly, RY2 exhibited specific down‐regulation of HPCA1, an extracellular ROS receptor, implying compromised ROS‐mediated immunity compared to 2001‐84.

To investigate phosphorylation dynamics in CDPKs and MAPKs, we identified 8 CDPKs, 1 MAPK kinase kinase (MAPKKK), 2 MAPKs and 6 regulatory phosphatases. The phosphorylation profiles revealed distinct patterns between genotypes: 4 CDPKs showed increased phosphorylation in RY2, whereas 5 CDPKs, 1 MAPKKK and both MAPKs were up‐regulated in 2001‐84. Notably, three CDPKs (CPK5, CPK10 and CPK28) exhibited up‐regulation in both genotypes but demonstrated higher induction in 2001‐84 (Figure S4, Table S12). Intriguingly, phosphatases responsible for CDPK and MAPK dephosphorylation were predominantly up‐regulated in RY2 but remained largely unchanged in 2001‐84 (Figure S4, Table S12). These results indicate more robust activation of CDPK‐ and MAPK‐regulated signalling pathways in 2001‐84 compared to RY2.

In addition to receptors, transporters represented a major class of plasma membrane proteins identified in our analysis. We therefore characterised their phosphorylation patterns in response to pathogen infection. A total of 19 transporters were identified, including 2 calcium channels, 10 ABC transporters, 2 boron transporters, 3 potassium transporters, 1 malate transporter and 1 inositol transporter. Notably, pathogen infection induced widespread dephosphorylation of these transporters, with 13 transporters down‐regulated in RY2 compared to only 7 in 2001‐84. Interestingly, while 4 transporters showed consistent down‐regulation in both genotypes, 9 exhibited the suppression exclusively in RY2 (Figure S4, Table S12). These findings suggest that pathogen‐induced dephosphorylation of transporters may represent a virulence strategy to facilitate infection, with RY2 showing greater susceptibility than 2001‐84.

Because the DPPs between 2001‐84 and RY2 at 96 hpi were significantly enriched in oxidoreductase activity in molecular functions (Figure 3a, Table S9), we further analysed oxidoreductase‐related proteins and their phosphorylation patterns. Among the 8 up‐regulated DPPs associated with oxidoreductase activity in response to pathogen infection, 2 were common to both genotypes, while 3 were uniquely regulated in each genotype (Figure S4, Table S12). These findings suggest that pathogen infection induces phosphorylation of redox‐regulatory proteins in both genotypes, albeit with genotype‐specific differences.

### 2.4 Plasma Membrane Proteomic Analysis of RY2 and 2001‐84 in Response to *C. gloeosporioides*

The phosphoproteomic results showed that the DPPs in RY2 and 2001‐84 were both significantly enriched in the plasma membrane (Figure 3b), indicating that plasma membrane (PM) proteins play a crucial role in the resistance of stylo to *C. gloeosporioides*. Therefore, the leaf samples collected at the same time points as for phosphoproteomics were enriched for PM proteins (Figure S5a). The PM‐enriched fractions were used for proteomics analysis to identify differentially accumulated PM proteins (DAPs) in RY2 and 2001‐84 responding to inoculation. The PCA results showed that the samples at 0 and 96 hpi were significantly separated by PC1 and PC2, with good repeatability within three biological replicates, indicating that the data was reliable and could be further analysed (Figure S5b). Comparative analysis between 2001‐84 and RY2 identified 36 and 54 DAPs at 0 hpi (CK) and 96 hpi, respectively (Figure S5c, Tables S13 and S14). Temporal analysis comparing 96 hpi with 0 hpi revealed 114 DAPs in RY2 and 133 DAPs in 2001‐84 (Figure S4c, Tables S15 and S16), demonstrating dynamic accumulation changes during pathogen infection. Consistent with the phosphoproteomic analysis, we examined the pathogen‐induced accumulation dynamics of PM‐localised receptor and transporter proteins, which constituted the major group identified in DAPs. The analysis identified 22 receptors and 18 transporters showing differential accumulation. In RY2, 9 receptors and 3 transporters were up‐regulated, whereas 4 receptors and 4 transporters were down‐regulated. In 2001‐84, 15 receptors and 13 transporters were up‐regulated, whereas 1 receptor and 2 transporters were down‐regulated. Interestingly, 1 ABC transporter, 4 aquaporins, 2 potassium transporters, 2 inositol transporters and 1 auxin transporter were exclusively induced in 2001‐84 (Figure S6, Table S17). Given that the identified receptors and transporters are functionally implicated in pathogen perception, defence signal transduction, and nutrient transport, these results indicate that the higher induction of these proteins in 2001‐84 may contribute to the enhanced resistance compared to RY2.

### 2.5 Identification of Protein Candidates That May Regulate Resistance Against *C. gloeosporioides*

**FIGURE 4:**
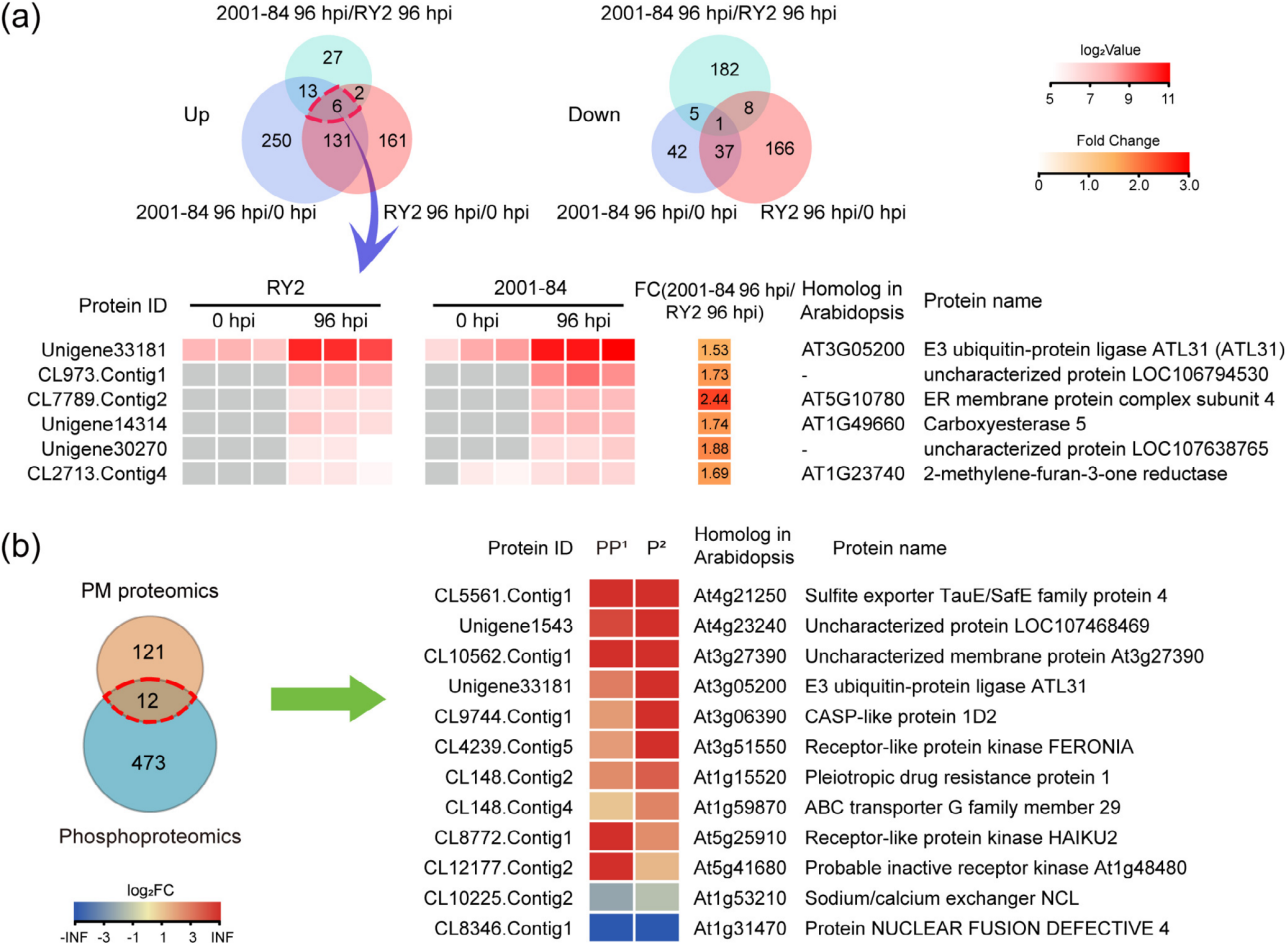
Integrated analysis of resistance‐associated proteins identified through phosphoproteomic and plasma membrane (PM) proteomic profiling. (a) Comparative analysis of pathogen‐induced differentially phosphorylated proteins (DPPs). Venn diagrams showing unique and shared up‐/down‐regulated DPPs among three comparison groups: 2001‐84 (96 h post‐inoculation [hpi] vs. 0 hpi), RY2 (96 hpi vs. 0 hpi) and 2001‐84 vs. RY2 at 96 hpi. Heatmap displays log_2_‐transformed phosphorylation levels of DPPs. The fold change (FC) represents the ratio of average phosphorylation levels between 2001‐84 and RY2 at 96 hpi. (b) Overlap analysis between phosphoproteomic and PM proteomic datasets in 2001‐84. Venn diagram comparing pathogen‐responsive DPPs and differentially accumulated proteins (DAPs) in 2001‐84. Heatmap shows: PP^1^, log_2_FC of DPPs (96 hpi/0 hpi); P^2^: log_2_FC of DAPs (96 hpi/0 hpi).

To identify proteins of stylo potentially regulating the resistance against *C. gloeosporioides*, all pathogen‐induced DPPs in RY2 and 2001‐84 from phosphoproteomics were analysed by Venn diagram and heatmap analysis. The results showed that 6 DPPs were up‐regulated after inoculation with *C. gloeosporioides* in both RY2 and 2001‐84, and the phosphorylation level was further increased in 2001‐84 compared to RY2 (Figure 4a). Examining both PM proteomic and phosphoproteomic datasets, only 12 pathogen‐responsive proteins were identified in both omics in 2001‐84, among which 10 proteins were up‐regulated after inoculation (Figure 4b). As suggested by phosphoproteomics and PM proteomics, CDPK activity, oxidoreductase activity and membrane transport may contribute to the resistance against *C. gloeosporioides*. AtATL31/6 has been previously reported to be involved in regulating CDPKs and ROS during *Arabidopsis–*pathogen interaction as well as nutrient transport (Maekawa et al. 2014; Liu et al. 2022). SgATL31 was identified in both omics and accumulated to higher levels in the resistant accession 2001‐84 responding to *C. gloeosporioides* infection. Therefore, SgATL31 was selected for further investigation as a potential regulator contributing to the resistance against *C. gloeosporioides*.

### 2.6 SgATL31 Localises to the Plasma Membrane and Exhibits Ubiquitination Activity

**FIGURE 5:**
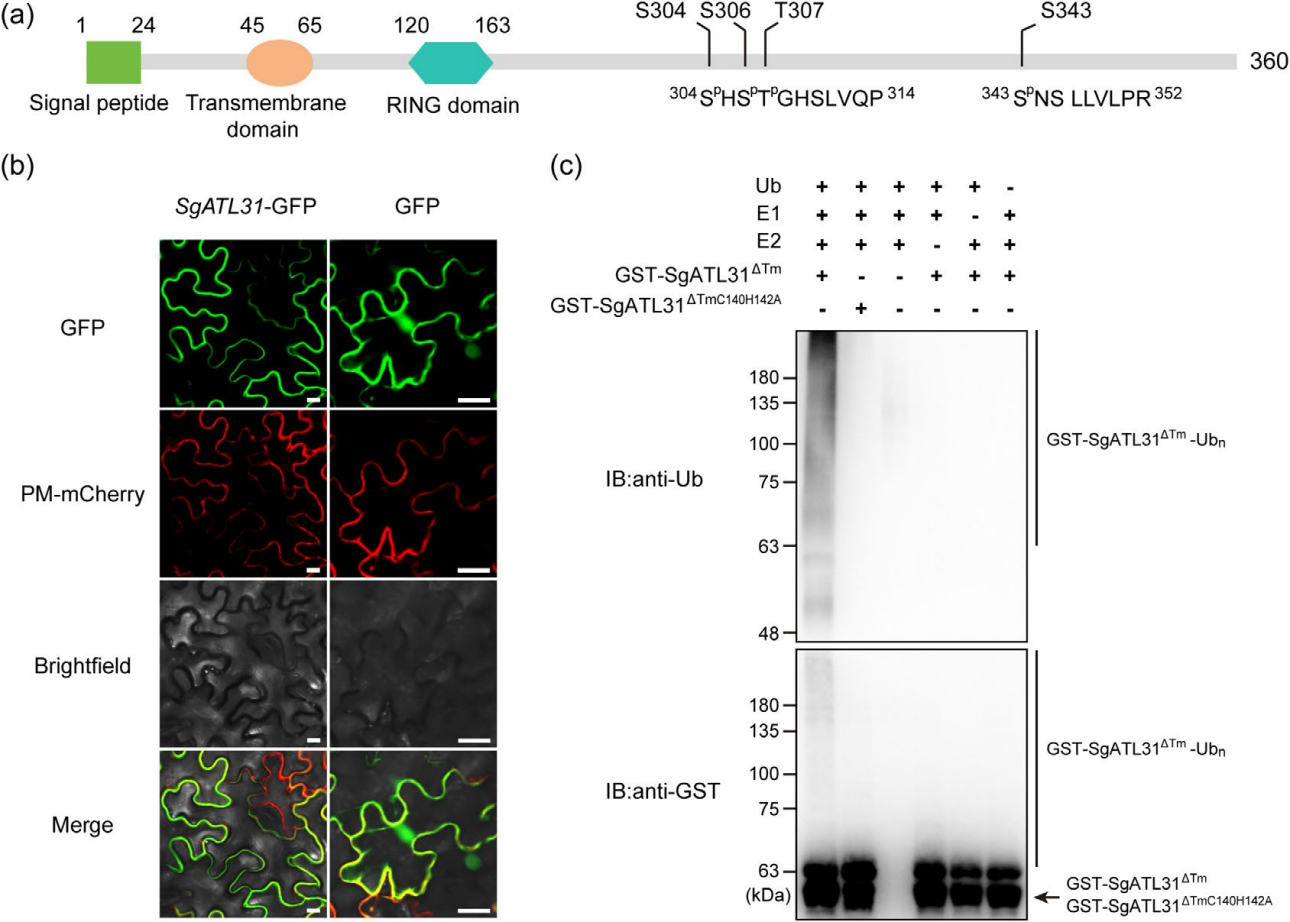
Subcellular localisation and in vitro ubiquitination assay of SgATL31 protein. (a) The conserved domain analysis of SgATL31. Green, orange and blue indicate signal peptide, transmembrane domain and RING domain, respectively. S304, S306, T307 and S343 represent the residues differentially phosphorylated in response to *Colletotrichum gloeoporioides*. (b) The subcellular localisation of SgATL31 in *Nicotiana benthamiana*. Bars, 20 μm. (c) In vitro ubiquitination assay. The reactions were performed by combining purified GST‐SgATL31^∆Tm^ (and its RING finger mutant GST‐SgATL31^∆TmC140H142A^) with the Ub, E1 and E2 enzymes. The ubiquitination signal was detected using Ub antibody (top panel). The presence of recombinant protein in the assay was detected using GST antibody (bottom panel, arrow shows target protein bands). E1, human UBE1; E2, human UBE2N/UBE2V2 complex; GST‐SgATL31^∆Tm^, 59 kDa; GST‐SgATL31^∆TmC140H142A^, 58.9 kDa.

To characterise SgATL31, we analysed its coding sequence (CDS) through sequence alignment, phylogenetic reconstruction and domain prediction. The amplified sequences from both 2001‐84 and RY2 matched published RY2 transcriptome data (Figure S7a). Phylogenetically, SgATL31 clustered with legume ATL31s, showing the highest homology to *Arachis* species (*A. ipaensis*, *A. duranensis* and *A. hypogaea* ). Motif analysis revealed 10 conserved motifs shared across legume ATL31s (*Stylosanthes*, *Medicago*, *Trifolium*, *Cicer*, *Glycine*, *Vigna* and *Arachis*), underscoring its evolutionary conservation (Figure S7b). Structurally, SgATL31 harbours a signal peptide, transmembrane domain and RING domain, suggesting its identity as a PM‐localised RING‐type E3 ligase. Notably, pathogen‐induced phosphorylation sites mapped to its C‐terminus (Figure 5a). Subcellular localisation assays using a SgATL31‐GFP fusion confirmed PM targeting, as evidenced by colocalisation with PM‐mCherry (Figure 5b), consistent with in silico predictions.

To assess the ubiquitin ligase activity of SgATL31, we performed an in vitro ubiquitination assay using GST‐tagged SgATL31^∆Tm^ (lacking the transmembrane domain) and a RING finger mutant (SgATL31^∆TmC140H142A^), in which the conserved Cys140 and His142 were substituted with Ala. Immunoblot analysis with an anti‐ubiquitin antibody revealed that GST‐SgATL31^∆Tm^ catalysed the formation of high‐molecular‐weight polyubiquitin chains, indicating its ubiquitin ligase activity (Figure 5c). In contrast, no activity was detected in the RING mutant (SgATL31^∆TmC140H142A^) or when any key component (E1, E2, Ub or SgATL31) was omitted from the reaction. The autoubiquitination of GST‐SgATL31^∆Tm^, along with equal loading of GST‐fusion proteins, was verified by anti‐GST immunoblotting (Figure 5c). These results demonstrate that SgATL31 functions as a RING‐type E3 ubiquitin ligase, with its activity dependent on an intact RING finger domain.

### 2.7 Overexpression of *SgATL31* Increases Resistance Against *C. gloeosporioides*

**FIGURE 6:**
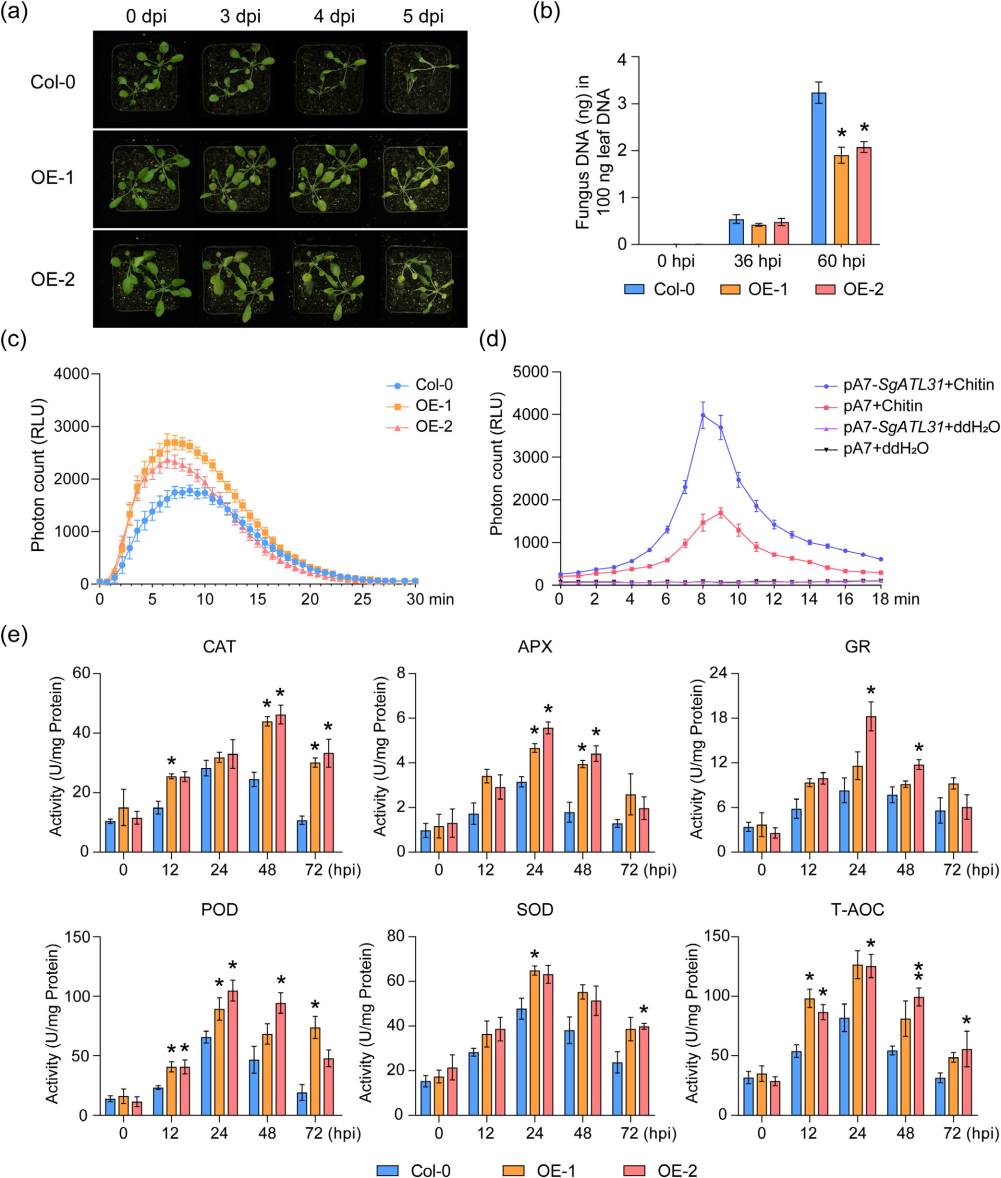
Functional characterisation of *SgATL31*. (a) The disease phenotypes of *SgATL31* overexpression lines and wild type *Arabidopsis* after inoculation with *Colletotrichum gloeosporioides*. (b) The accumulation of *C. gloeosporioides* in Col‐0 and OE‐1/2 at 0, 36, 60 h post‐inoculation (hpi). (c) The detection of chitin‐induced reactive oxygen species (ROS) in Col‐0 and OE‐1/2 lines. Data are means ± SE pooled from three replicates (*n* = 12). (d) The detection of chitin‐induced ROS in stylo protoplasts. Data are means ± SE pooled from three replicates (*n* = 6). (e) The detection of the activity of catalase (CAT), ascorbate peroxidase (APX), glutathione reductase (GR), peroxidase (POD), superoxide dismutase (SOD), total antioxidant capacity (T‐AOC) in Col‐0 and OE‐1/2 at 0, 12, 24, 48, 72 hpi. OE‐1/2, *Arabidopsis* lines overexpressing *SgATL31*. Data are means ± SE pooled from three replicates (*n* = 3). Statistical significance is assessed in Student's *t* tests. Significant differences are observed between Col‐0 and OE‐1/2 (*p < 0.05, **p < 0.01).

To examine the function of SgATL31 in disease resistance, two transgenic *Arabidopsis* lines (OE‐1/2) overexpressing *SgATL31* were generated (Figure S8). The pathogen assays were performed using 4‐week‐old plants by spray‐inoculation of spore suspension of *C. gloeosporioides*. The results showed that the severity of disease symptoms was milder in OE‐1/2 compared to wild‐type plants (Col‐0), and the disease progression was also slower in OE‐1/2 (Figure 6a). Consistently, the result of qPCR analysis showed that the amount of *C. gloeosporioides* DNA in OE‐1/2 was significantly lower than that in Col‐0 at 60 hpi (Figure 6b). These results indicated that overexpression of *SgATL31* significantly enhanced the resistance against *C. gloeosporioides*.

### 2.8 Overexpression of *SgATL31* Increases Chitin‐Induced ROS Burst and Antioxidant Enzyme Activities Responding to *C. gloeosporioides* Infection

Because AtATL31/6 has been previously shown to positively regulate the PAMP‐triggered ROS burst and resistance in *Arabidopsis* (Liu et al. 2022), the ROS production of OE‐1/2 was dynamically monitored after chitin treatment. The results showed that the OE‐1/2 produced significantly higher ROS than Col‐0 (Figure 6c). In addition, transient expression of *SgATL31* in stylo protoplasts also showed that overexpression of *SgATL31* increased the chitin‐triggered ROS burst (Figure 6d). These results suggest that *SgATL31* positively regulates the chitin‐triggered ROS burst, resulting in enhanced resistance.

The ROS burst could induce the antioxidant defence systems, which is a prominent element in plant responses to fungal attack. Therefore, the activities of antioxidant enzymes CAT, ascorbate peroxidase (APX), GR, POD, SOD and T‐AOC were examined in OE‐1/2 and Col‐0 after *C. gloeosporioides* inoculation. The activities of CAT, APX, GR, POD, SOD and T‐AOC were all induced in both Col‐0 and OE‐1/2 in response to *C. gloeosporioides* infection. Interestingly, in OE‐1/2 lines, the activities of these antioxidant enzymes were not significantly different from Col‐0 at 0 hpi but induced at higher levels from 12 hpi to 72 hpi (Figure 6e). These results indicate that higher levels of antioxidant activities may also contribute to the resistance against *C. gloeosporioides*.

### 2.9 SgATL31 Exhibits Distinct Protein Interaction Specificity Compared to AtATL31

In *Arabidopsis*, AtATL31 confers resistance to powdery mildew by interacting with the PM‐localised protein AtSYP121 (Maekawa et al. 2014) and enhances immunity by proteasomal degradation of AtCPK28 (Liu et al. 2022). To investigate whether SgATL31 maintains the conserved protein interactions of its *Arabidopsis* orthologue AtATL31, we performed bimolecular fluorescence complementation (BiFC) assays in both *Nicotiana benthamiana* and *Arabidopsis* protoplast systems. Our analysis included SgATL31 (and its RING mutant, SgATL31^C140H142A^) and three candidate interactors: AtCPK28, SgCPK28 and SgSYP121. As a positive control, we confirmed the known interaction between AtATL31 (and its RING mutant, AtATL31^C143H145A^) and AtCPK28 in both systems. However, no interaction was detected between SgATL31 and any of the tested proteins (Figures S9 and S10). These results imply that SgATL31 may engage different signalling pathways by associating with distinct partners compared to AtATL31.

### 2.10 Expression Patterns of *SgATL31* in 40 Stylo Accessions After Inoculation

**FIGURE 7:**
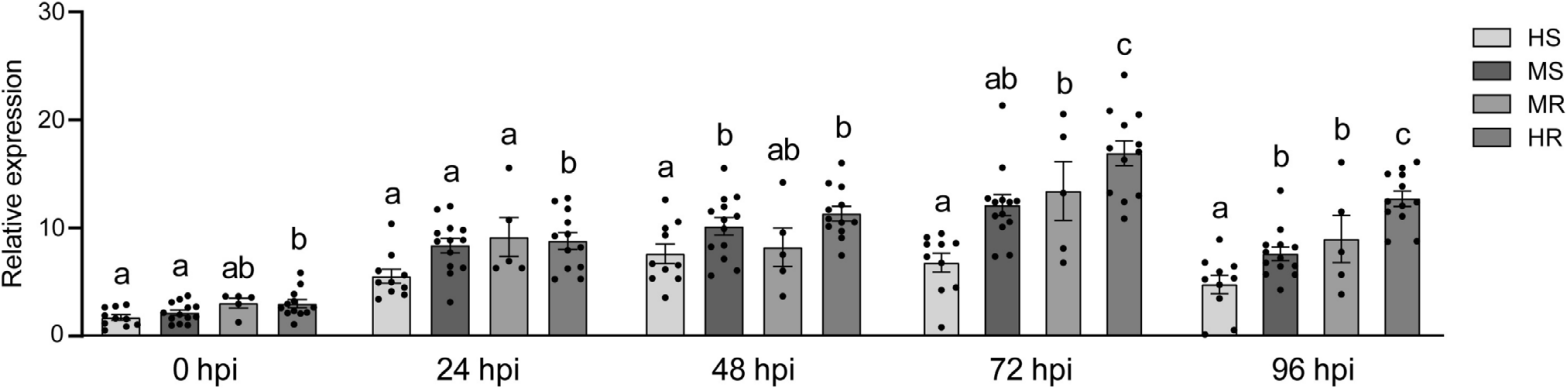
The expression pattern of *SgATL31* in 40 stylo accessions after inoculation. HS, MS, MR, and HR indicate highly susceptible, moderately susceptible, moderately resistant, and highly resistant, respectively. Data are means ± SE pooled from three replicates (*n* = 3). Statistical significance is assessed in one‐way ANOVA (*p* < 0.05).

To explore the correlation between the expression levels of *SgATL31* and disease resistance in 40 stylo accessions, the expression patterns of *SgATL31* in 40 stylo accessions inoculated with *C. gloeosporioides* were analysed. The results showed that the expression levels of *SgATL31* in 40 stylo accessions were significantly induced after inoculation and accumulated at a higher level in resistant accessions at 72 hpi and 96 hpi, indicating that a higher expression level of *SgATL31* may contribute to the higher resistance (Figure 7).

## 3 Discussion

Anthracnose is a serious fungal disease affecting stylo production. However, disease incidence and severity vary among different stylo accessions (Cameron et al. 1978). In this study, we systematically evaluated the resistance traits of 40 stylo accessions based on five key indexes, with the commercially predominant cultivar RY2 serving as the control. The comprehensive and discriminant analysis identified 2001‐84 as the most resistant accession. Comparative phenotypic analysis revealed that 2001‐84 significantly delayed disease progression and restricted *C. gloeosporioides* infection compared to RY2. Additionally, 2001‐84 demonstrated elevated pathogen‐induced antioxidant enzyme activity, suggesting enhanced oxidative stress management during infection. To explore the molecular basis of resistance, we performed comparative phosphoproteomic and PM‐enriched proteomic analyses between 2001‐84 and RY2, focusing on the biotrophic‐to‐necrotrophic transition and necrotrophic phases of infection. The results showed that the pathogen‐induced differentially phosphorylated/expressed proteins were mainly enriched in protein kinase, transporter, and oxidoreductase activity. Elucidating the roles and expression dynamics of these proteins in resistant (2001‐84) and susceptible (RY2) genotypes provides critical insights into the molecular mechanisms regulating stylo resistance to *C. gloeosporioides*.

RLKs, CDPKs and MAPKs are central to immune signal perception and transduction via phosphorylation cascades (Wang et al. 2024). PAMP/DAMP recognition triggers RLK phosphorylation, initiating downstream events including Ca^2+^ influx, ROS burst and CDPK/MAPK activation, ultimately regulating defence‐related gene expression (Jones et al. 2024). During *C. gloeosporioides* infection, RLK and CDPK phosphorylation increases in stylo throughout the biotrophic–necrotrophic transition, suggesting their role in defence signal amplification. Notably, 2001‐84 shows exclusive up‐regulation of SgMAPKKK3, SgMPK9 and SgMPK6 phosphorylation, along with stronger induction of SgCPK5/10/28 and more RLK up‐regulation than RY2. These findings demonstrate enhanced RLK‐, CDPK‐ and MAPK‐mediated immune activation in 2001‐84, potentially explaining its superior resistance.

Transporters play pivotal roles in plant immunity by regulating the exchange of ions, metabolites and signalling molecules. ABC transporters mediate defence compound efflux (Devanna et al. 2021), calcium transporters shape Ca^2+^ signatures for immune signalling (Wang et al. 2024), and aquaporins (AQPs) modulate H_2_O_2_ diffusion to fine‐tune ROS homeostasis (Mittler et al. 2022). Their activity is often regulated by phosphorylation, for instance, AtABCG36 phosphorylation by QSK1 promotes camalexin export (Aryal et al. 2023), and BIK1‐mediated phosphorylation enhances AtOSCA1.3 channel activity for stomatal immunity (Thor et al. 2020). Pathogens exploit this regulatory layer by secreting phosphatases (e.g., *Ustilaginoidea virens*; Zheng et al. 2022) or hijacking host phosphatases (e.g., *Phytophthora* effectors; Li et al. 2023) to dephosphorylate transporters, suppressing defences. Our data align with this strategy: phosphoproteomics revealed widespread transporter dephosphorylation in susceptible RY2, while resistant 2001‐84 maintained phosphorylation for 9 out of 13 affected transporters. Furthermore, PM proteomics showed 2001‐84 uniquely up‐regulates 10 transporters (including ABC, AQPs and nutrient transporters) post‐infection, compared to only 3 in RY2. This coordinated retention of phosphorylation and broader transporter induction likely underpins 2001‐84's enhanced resistance.

Plants employ antioxidant systems to maintain ROS homeostasis, preventing oxidative damage while supporting immune responses, a critical defence against hemibiotrophic and necrotrophic pathogens (Cao et al. 2024; do Carmo Santos et al. 2024). Resistant accession 2001‐84 showed significantly elevated activities of key antioxidant enzymes (CAT, T‐SOD, CuZn‐SOD, T‐AOC, POD, GR) during *C. gloeosporioides* infection compared to susceptible RY2, suggesting enhanced ROS‐scavenging capacity contributes to its resistance. Our phosphoproteomic analysis also revealed differential regulation of ROS‐scavenging proteins between the two cultivars. While two phosphoproteins (SgGR1 and SgCAT2) were commonly up‐regulated in both genotypes, three were uniquely induced in each (SgGSTF9, SgOXR, SgNDHJ in RY2; SgCXXS1, SgGPX6, SgGST in 2001–84), suggesting genotype‐specific antioxidant strategies. Interestingly, studies in both plant and mammal have shown that phosphorylation is an effective mechanism regulating the antioxidant enzyme activities (Zhou et al. 2019; He et al. 2024). Therefore, our studies also indicate that phosphorylation‐mediated activation of ROS‐scavenging enzymes represents a defence mechanism of stylo against *C. gloeosporioides*.

Integrated phosphoproteomic and PM proteomic analyses identified SgATL31 as a potential regulator of resistance against *C. gloeosporioides* in stylo. ATL family proteins are well documented in plant immunity, such as *Arabidopsis* AtATL31/6 against *Pseudomonas syringae* and powdery mildew (Maekawa et al. 2012, 2014; Liu et al. 2022), potato StRFP1/NbATL60 against *Phytophthora infestans* (Zhong et al. 2018), and rice OsBIRF1 against viral and bacterial pathogens (Liu et al. 2008). However, their role in hemibiotrophic fungal resistance remains unexplored. Here, we demonstrate that SgATL31 enhances resistance to *C. gloeosporioides*. Overexpressing *SgATL31* in *Arabidopsis* conferred increased pathogen resistance, mirroring findings in other ATLs. Notably, SgATL31 induction was stronger in the resistant genotype 2001‐84 than in the susceptible RY2, and its expression correlated with resistance across accessions. These results establish SgATL31 as a positive regulator of defence against *C. gloeosporioides*.

Overexpression of *SgATL31* enhanced chitin‐induced ROS burst and antioxidant enzyme activities during *C. gloeosporioides* infection. We propose SgATL31 confers resistance by balancing ROS production and antioxidant defences. While ROS strengthens cell walls and mediates immune signalling, excessive ROS causes oxidative damage, necessitating antioxidant regulation (Mittler et al. 2022; Wu, Qi, and Liang 2023). For example, *C. gloeosporioides* infection elevated both antioxidant enzymes (SOD, CAT, POD, GPX, GR) and compounds (ascorbic acid, glutathione) in stylo leaves (Wang et al. 2017; Zhang et al. 2024). Similarly, the ATL subfamily member PbATL18 boosted pear resistance to *Colletotrichum fructicola* by enhancing PAL, CAT, polyphenol oxidase and chitinase activities while maintaining substrate homeostasis (Lin et al. 2024). In this study, the activity of antioxidant enzymes, CAT, APX, GR, POD, SOD and T‐AOC, in the SgATL31 OE lines was significantly higher than that in Col‐0 after *C. gloeosporioides* inoculation, especially at the later stage of inoculation. Therefore, these results showed that SgATL31 inhibited the infection of *C. gloeosporioides* by producing higher ROS during the early stage of inoculation, which triggered higher activities of antioxidant enzymes to maintain the homeostasis of ROS in the late stages, thereby enhancing the resistance against *C. gloeosporioides*.

As E3 ligases, ATL proteins mediate ubiquitination of target substrates, leading to proteasomal degradation or functional modulation (Chen and Hellmann 2013). Identifying these substrates is crucial for understanding their defence mechanisms. In *Arabidopsis*, AtATL31 interacts with PM‐localised AtSYP121 to promote papilla formation during powdery mildew infection (Maekawa et al. 2014), and targets AtCPK28 for degradation to enhance immunity (Liu et al. 2022). Although we cloned *Arabidopsis* AtCPK28 and orthologue proteins of AtSYP121 and AtCPK28 in stylo, BiFC assays in *N. benthamiana* and *Arabidopsis* protoplasts revealed no interaction with SgATL31. Given that substrate specificity is determined by the C‐terminal region following the RING‐H2 domain, which differs between SgATL31 and AtATL31, SgATL31 likely recognises distinct substrates. Future studies should focus on identifying SgATL31's specific interaction partners to elucidate its resistance mechanisms.

**FIGURE 8:**
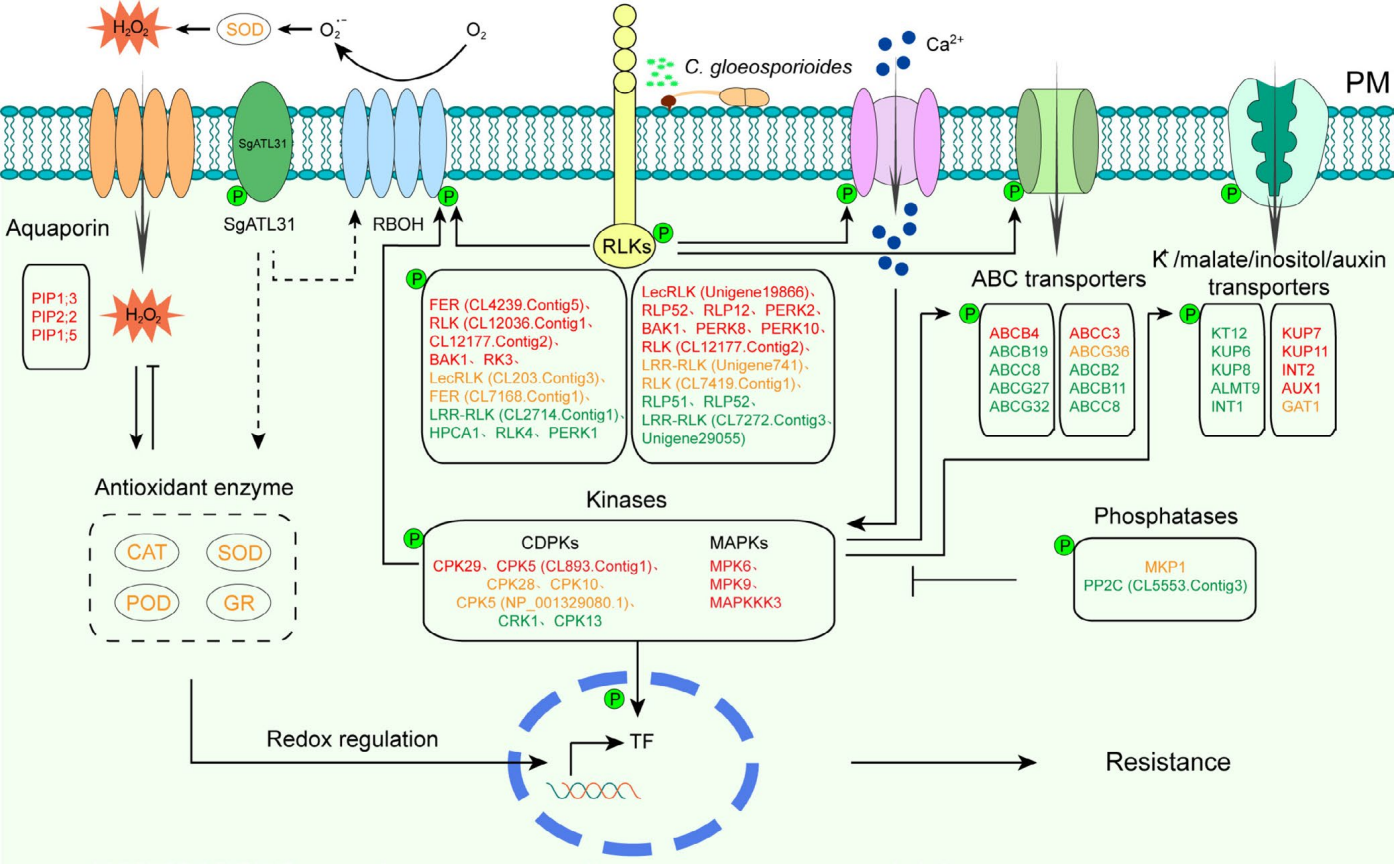
Proposed molecular mechanism of anthracnose resistance in the resistant accession 2001‐84 versus susceptible cultivar RY2. Upon *Colletotrichum gloeosporioides* infection, 2001‐84 exhibits broader induction of calcium‐dependent protein kinase (CDPK)/mitogen‐activated protein kianse (MAPK) phosphorylation and receptor‐like kinase (RLK)/transporter up‐regulation, reduced transporter dephosphorylation, enhanced antioxidant activity and increased SgATL31 phosphorylation and accumulation, promoting reactive oxygen species (ROS) burst and antioxidant activation. Genotype‐specific proteins responding to pathogen infection are indicated in the solid‐lines boxes identified from phosphoproteomics (P‐marked) and plasma membrane enriched proteomics (unmarked). The experimentally validated antioxidant enzymes are indicated in the dashed‐line boxes. Proteins in red are specifically up‐regulated in 2001‐84. Proteins in orange are up‐regulated in both genotypes with greater fold‐change in 2001‐84 than in RY2. Proteins in green are down‐regulated in RY2 but not significantly changed in 2001‐84.

Based on our integrated findings, we propose a mechanistic model underlying anthracnose resistance in the resistance accession 2001‐84 (Figure 8). Compared to the susceptible cultivar RY2, 2001‐84 demonstrates broader induction of CDPK and MAPK phosphorylation, reduced susceptibility to transporter dephosphorylation, enhanced up‐regulation of RLKs and transporters, and superior antioxidant activity upon *C. gloeosporioides* infection. Furthermore, 2001‐84 shows elevated SgATL31 phosphorylation and protein accumulation, which contribute to disease resistance by promoting a stronger ROS burst and stimulating antioxidant enzyme activity. These synergistic mechanisms collectively enhance anthracnose resistance in 2001‐84. Importantly, the observed protein differences may represent pleiotropic effects resulting from mutations or allelic variations at secondary loci. In this study, the accession 2001‐84 demonstrated significantly enhanced resistance compared to the widely cultivated cultivar RY2, positioning it as an elite parental line for resistance breeding programmes. The integration of its superior resistance traits with marker‐assisted selection (MAS) and gene editing could facilitate simultaneous improvement of both resistance and key agronomic characteristics. To fully exploit this germplasm resource, future research should focus on fine‐mapping the causal genetic elements (such as quantitative trait loci [QTLs]) through the construction of biparental populations and the development of the anthracnose resistance markers to support precision breeding strategies.

## 4 Experimental Procedures

### 4.1 Plant Material and Growth Condition

The main cultivar RY2 stylo ( *Stylosanthes guianensis* ) and 39 stylo accession resources (2001‐2, 2001‐3, 2001‐4, 2001‐6, 2001‐7, 2001‐11, 2001‐15, 2001‐16, 2001‐17, 2001‐19, 2001‐20, 2001‐22, 2001‐24, 2001‐25, 2001‐27, 2001‐28, 2001‐34, 2001‐40, 2001‐42, 2001‐44, 2001‐47, 2001‐54, 2001‐57, 2001‐59, 2001‐62, 2001‐63, 2001‐64, 2001‐66, 2001‐69, 2001‐71, 2001‐73, 2001‐75, 2001‐76, 2001‐77, 2001‐78, 2001‐79, 2001‐80, 2001‐81, 2001‐84) were used. The stylo accessions were provided by Tropical Crops Genetic Resources Institute, Chinese Academy of Tropical Agricultural Sciences (CATAS), derived from mutagenesis and selective breeding of progeny populations originating from CIAT184, the germplasm resources initially introduced from the International Center for Tropical Agriculture (CIAT). The seed germination and growth procedures were described previously (Yang et al. 2022). The growth temperature of stylo ranged from 25°C to 32°C and relative humidity from 60% to 80%. *N. benthamiana* and *Arabidopsis* were grown in a 22°C greenhouse with a 16 h/8 h light/dark photoperiod cycle.

### 4.2 Inoculation of *C. gloeosporioides*

*Colletotrichum gloeosporioides* was cultured and inoculated using methods described in previous studies (Wang et al. 2017). 4‐week‐old plants of stylo and *Arabidopsis* were inoculated with 1 × 10^7^ conidia/mL and 5 × 10^6^ conidia/mL spore suspension containing 0.02% Silwet L‐77 (Solarbio), respectively.

### 4.3 Evaluation of Stylo Accessions for the Resistance to *C. gloeosporioides*

Resistance evaluation was conducted using established methods (Chakraborty 1990; Wan et al. 2022). Five disease parameters were assessed: leaf disease severity (LRAT), stem disease severity (SRAT), percentage defoliation (DEFL), lesion area per leaf (ALES) and dry weight index (DRWT). Detailed assessment methodology and analysis are provided in Supporting Information.

### 4.4 Microscopic Observation of *C. gloeosporioides* Infection in Stylo Leaves

Leaf samples of stylo accessions RY2 and 2001‐84 were collected at different time points after inoculation with *C. gloeosporioides* and stained following Jiang et al. (2021). Briefly, leaves were fixed in FAA (formalin‐acetic acid‐alcohol) solution for 15 min, decolourised in saturated chloral hydrate solution (Macklin), and stained with 0.5% aniline blue for 15 min. Fungal structures were then examined under a DM2000 optical microscope (Leica).

### 4.5 Reverse transcription‐qPCR

Total RNA was extracted with TRIzol Universal reagent (TIANGEN) as previously described (Yang et al. 2022). Briefly, first cDNA strand was synthesised from 1 μg RNA using HiScript II 1st Strand cDNA Synthesis Kit (+gDNA wiper); Vazyme. qPCR was performed by using ChamQ Universal SYBR qPCR Master Mix (Vazyme) following standard protocols on Applied Biosystems QuantStudio 7 Flex System (Applied Biosystems). *UBCE1* (ubiquitin‐conjugating enzyme 1) was used as an internal reference gene to normalise gene expression (Jiang et al. 2021) (Table S18). A total of three biological replicates were used to calculate relative expression levels using 2^−∆∆Ct^ (Schmittgen and Livak 2008).

### 4.6 Determination of *C. gloeosporioides* DNA in Stylo and *Arabidopsis* Leaves

The DNA levels of *C. gloeosporioides* in stylo and *Arabidopsis* leaves were quantified at different time points using a previously described method (Wan et al. 2022). Briefly, genomic DNA was extracted using the CTAB (cetyltrimethylammonium bromide) method. The extracted DNA served as the template for qPCR with the following primers: *C. gloeosporioides ACT4* (ACT4‐qPCR‐F/R), stylo *SgUBCE1* (SgUBCE1‐qPCR‐F/R), *Arabidopsis QACT* (QACT‐qPCR‐F/R) (Table S18). The *C*_t_ values obtained were used to calculate fungal and plant DNA content based on the standard curve established by Wan et al. (2022).

### 4.7 Determination of Antioxidant Enzyme Activity

The SOD, POD, CAT, T‐AOC, GR and APX assay kit (Nanjing Jiancheng Bioengineering Institute) were used to determine the antioxidant enzyme activity in the leaves of stylo and *Arabidopsis* after being infected with *C. gloeosporioides*.

### 4.8 Proteomic Analysis

Phosphoproteomic and PM‐enriched proteomic analyses were performed by BGI Tech Solutions Co. Ltd. (Shenzhen, China). Detailed methods for protein isolation, phosphopeptide/peptide enrichment, liquid chromatography‐tandem mass spectrometry (LC–MS/MS) analysis, and protein identification are provided in Supporting Information. Protein identification used the published stylo transcriptome (Jiang et al. 2021) as reference, with quality control by mProphet algorithm. Phosphosites were filtered at probability ≥ 0.75, and identifications were validated at false discovery rate (FDR) ≤ 0.01 (Käll et al. 2007). Differential proteins were defined as those with fold change > 1.5 or < 0.67 and *p* < 0.05 (MSstats: https://bioconductor.org/packages/release/bioc/html/MSstats.html). Bioinformatics analyses included: PCA visualisation (ggplot2: https://ggplot2.tidyverse.org/), functional enrichment (GO/KEGG; clusterProfiler: https://www.bioconductor.org/packages/release/bioc/html/clusterProfiler.html), subcellular localisation prediction (WoLF PSORT: https://wolfpsort.hgc.jp/), heatmap generation (TBtools; Chen et al. 2023).

### 4.9 Subcellular Localisation Analysis

The SgATL31 coding sequence (CDS) was amplified from RY2 and 2001‐84 cDNA using transcriptome‐derived primers (Jiang et al. 2021; Table S18) and cloned into pA7‐SgATL31‐GFP and pCAMBIA2300‐SgATL31‐GFP vectors. For subcellular localisation, *Agrobacterium tumefaciens* GV3101 harbouring either pCAMBIA2300‐SgATL31‐GFP (test) or pCAMBIA2300‐GFP (control), together with the PM marker pEG100‐PIP2A‐mCherry (1:1 ratio, OD_600_ = 1.0 in 10 mM MES/MgCl_2_), were infiltrated into 4‐week‐old *N. benthamiana* leaves. Confocal microscopy (TCS SP8; Leica) was performed 48 h post‐infiltration using 488 nm (GFP) and 587 nm (mCherry) excitation wavelengths.

### 4.10 In Vitro Ubiquitination Assay

The truncated SgATL31 fragment (SgATL31^ΔTm^, 66–360) and the RING mutant of ATL31 containing substitution of both Cys140 and His142 to Ala (SgATL31^ΔTmC140H142A^) were cloned into pGEX‐6P‐3 vector, and introduced into competent cells of *Escherichia coli* BL21 (DE3) (Weidi Bio). Recombinant protein expression was induced with 0.5 mM isopropyl‐β‐D‐thiogalactoside in Luria Bertani broth for 4 h at 28°C, and was purified using glutathione beads (BEAVER). The in vitro ubiquitination assays were performed according to the Auto‐ubiquitinylation kit manual (Yeasen Biotechnology) with minor modifications. Each reaction (20 μL final volume) contained 2 μL 10× reaction buffer, 1 μL 10× E1 enzyme (human UBE1), 0.5 μL 20× E2 (human UBE2N/UBE2V2 complex), 4 μL 5× ubiquitin, 2 μL 10× Mg^2+^‐ATP solution and 25 μg E3 ligase enzyme (GST‐SgATL31^ΔTm^ or SgATL31^ΔTmC140H142A^ purified protein), adding double‐distilled water to the final volume. The mixture was incubated at 37°C for 30 min and terminated by the addition of 5× SDS‐PAGE sample buffer and incubation at 95°C for 10 min. The reaction products were resolved by 10% SDS‐PAGE and analysed by immunoblotting with anti‐Ub (Yeasen Biotechnology) and anti‐GST (TransGen Biotech) antibodies.

### 4.11 Overexpression of *SgATL31* in *Arabidopsis*

The SgATL31 CDS was cloned into pCXSN‐Myc‐SgATL31 for *Arabidopsis* transformation (Wu, Zhao, et al. 2023). Third‐generation homozygous transgenic lines were used for experiments. RT‐PCR confirmed overexpression using SgATL31‐specific primers (SgATL31‐OE‐F/R), with QACT (QACT‐qPCR‐F/R) as the internal reference (Table S18).

### 4.12 ROS Detection

For *Arabidopsis*, 4‐mm leaf discs were placed in a 96‐well white Costar flat‐bottom plate containing sterile water, which was removed after overnight incubation. A 100 μL reaction solution (containing 0.2 μM luminol, 20 μg/mL horseradish peroxidase, and 100 μg/mL chitin) was added, and chemiluminescence was dynamically monitored for 30 min using an Infinite M200 Pro microplate reader (Tecan). For stylo protoplasts, plasmids pA7‐SgATL31‐GFP and pA7‐GFP were transfected. After adding 100 μL protoplast suspension to 100 μL reaction solution, chemiluminescence signals were recorded every minute using the same microplate reader. Controls contained double‐distilled water instead of chitin. All experiments included three independent biological replicates. Detailed protocols for stylo protoplast isolation and transformation are provided in the Supporting Information.

### 4.13 Statistical Analysis

GraphPad Prism v. 8.0.2 was used to make bar graphs and IBM SPSS Statistics v. 20 software was used for one‐way ANOVA and Student's *t* test.

## Author Contributions

**Liyun Yang:** methodology, investigation, data curation, formal analysis, visualisation, writing – original draft preparation. **Yunpiao Long:** investigation, validation, writing – original draft preparation. **Mengze Gao:** investigation, validation. **Shizi Zhang:** investigation, validation. **Jing Gao:** investigation, validation. **Lijuan Luo:** resources, conceptualisation, supervision. **Lingyan Jiang:** conceptualisation, supervision, funding acquisition, writing – review and editing.

## Conflicts of Interest

The authors declare no conflicts of interest.

## Supporting information


**Table S1.** Principal component analysis of five indexes.

**Table S2.** The comprehensive index values, membership function values and disease resistance of 40 stylo accessions.

**Table S3.** Discriminant analysis based on cluster analysis.

**Table S4.** Differentially phosphoproteins (DPPs) identified between 2001–84 and RY2 at 0 hpi.

**Table S5.** DPPs identified between 2001–84 and RY2 at 96 hpi.

**Table S6.** DPPs between 96 hpi and 0 hpi identified in RY2.

**Table S7.** DPPs between 96 hpi and 0 hpi identified in 2001–84.

**Table S8.** GO enrichment analysis of DPPs between 2001–84 and RY2 at 0 hpi.

**Table S9.** GO enrichment analysis of DPPs between 2001–84 and RY2 at 96 hpi.

**Table S10.** GO enrichment analysis of DPPs between 96 hpi and 0 hpi in RY2.

**Table S11.** GO enrichment analysis of DPPs between 96 hpi and 0 hpi in 2001–84.

**Table S12.** The DPPs in the category of protein kinases, transporters and oxidoreductase.

**Table S13.** Differentially accumulation plasma membrane proteins (DAPs) identified between 2001–84 and RY2 at 0 hpi.

**Table S14.** DAPs identified between 2001–84 and RY2 at 96 hpi.

**Table S15.** DAPs identified between 96 hpi and 0 hpi in RY2.

**Table S16.** DAPs identified between 96 hpi and 0 hpi in 2001–84.

**Table S17.** The DAPs in the category of protein kinases and transporters.

**Table S18.** The information of primers used in the research.

**Figure S1.** Resistance evaluation of 40 stylo accessions against *Colletotrichum gloeosporioides*. (a) The leaf disease severity (LRAT) evaluated at 5 day post inoculation (dpi). (b) The stem disease severity (SRAT) evaluated at 5 dpi. (c) The percentage defoliation (DEFL) evaluated at 5 dpi. (d) The area of lesions/leaf (ALES) evaluated at 7 dpi. (e) The area of dry weight index (DRWT) evaluated at 7 dpi. Data are means ± SE pooled from three biological replicates (*n* = 27–30). Statistical significance was assessed in Student’s *t*‐tests. Asterisks represent significant differences between RY2 (control) and 39 stylo accessions (*p < 0.05, **0.001 ≤ *p* < 0.01, ***p < 0.001).

**Figure S2.** Correlation and discriminant analysis of five disease index (LRAT, SRAT, DEFL, ALES and DRWT). (a) Correlation analysis. Circle sizes represent the magnitude of Pearson’s correlation coefficients. Embedded numerical values show exact correlation coefficients. Colour intensity indicates direction (blue, positive correlations; red, negative correlations). Asterisks mark statistically significant correlations (***p < 0.001). (b) Discriminant analysis. Blue, Highly Resistant (HR) accessions; Green, Moderately Resistant (MR) accessions; Yellow, Moderately Susceptible (MS) accessions; Purple, Highly Susceptible (HS) accessions.

**Figure S3.** Phosphoproteomic profiling of 2001–84 and RY2 accession in response to *Colletotrichum gloeosporioides* infection. (a) Phosphosite multiplicity distribution. The percentage of single‐ (one site), double‐ (two sites), and triple‐phosphorylated (three sites) peptide are shown. (b) Phosphoamino acid distribution. The percentage of phosphorylation events on serine (S), threonine (T), and tyrosine (Y) residues are quantified. (c) Principal component analysis (PCA) of phosphorylation profiles. P0 and P4 indicate the samples from RY2 collected at 0 hpi and 96 hpi, respectively. R0 and R4 indicate the samples from 2001–84 collected at 0 hpi and 96 hpi, respectively. Biological triplicates are represented by same‐coloured dots (*n* = 3 per condition). (d) The number of differentially phosphoproteins (DPPs). Colour codes indicate the number of DPPs in different comparison groups: Green, genotypic differences at 0 hpi (2001–84 vs. RY2); Blue, genotypic differences at 96 hpi (2001–84 vs. RY2); Orange, RY2 temporal changes (96 hpi vs. 0 hpi); Pink, 2001–84 temporal changes (96 hpi vs. 0 hpi).

**Figure S4.** Heatmap analysis of differentially phosphoproteins (DPPs) in RY2 and 2001–84 during *Colletotrichum gloeosporioides* infection revealed by phosphoproteomics. The heatmap displays DPPs belonging to the category of Protein kinases, Phosphatases, Transporters and Oxidoreductases. The protein kinases include receptor‐like kinase (RLK), calcium‐dependent protein kinase (CDPK), and mitogen‐activated protein kinase (MAPK). The transporters include Ca^2+^ transporter, ABC transporter, Boron transporter, Potassium transporter, Malate transporter and Inositol transporter. Red arrows: Significant upregulation (fold change > 1.5, *p* < 0.05); Blue arrows: Significant downregulation (fold change < 0.67, *p* < 0.05); ns: Not significant. Value represents the abundance of phosphoprotein calculated based on the peak intensities of the peptides.

**Figure S5.** Plasma membrane‐enriched proteomic profiling of 2001–84 and RY2 in response to *Colletotrichum gloeosporioides* infection. (a) Immunoblot analysis of subcellular fractions using organelle‐specific antibodies. Subcellular fractions: T, total homogenate; Cyto, cytosolic fraction; CM, crude microsomal fraction; PM, plasma membrane‐enriched fraction. Antibodies: AHA (H^+^‐ATPase, PM marker), AOX1/2 (mitochondrial marker). Total protein loads are detected by coomassie blue staining (CBB). Data represent three biological replicates collected from RY2 and 2001–84 at 0 and 96 h post inoculation (hpi). (b) Principal component analysis (PCA) of PM enriched proteomes. P0 and P4 indicate the samples from RY2 collected at 0 and 96 hpi, respectively. R0 and R4 indicate the samples from 2001–84 collected at 0 and 96 hpi, respectively. Biological triplicates are represented by same‐coloured dots (*n* = 3 per condition). (c) Differential accumulation of PM proteins. Colour codes indicate the number of differentially accumulated PM proteins (DAPs) in different comparison groups: Green, Genotypic differences at 0 hpi (2001–84 vs. RY2); Blue, Genotypic differences at 96 hpi (2001–84 vs. RY2); Orange, RY2 temporal changes (96 hpi vs. 0 hpi); Pink, 2001–84 temporal changes (96 hpi vs. 0 hpi).

**Figure S6.** Heatmap analysis of differentially accumulated proteins (DAPs) in RY2 and 2001–84 during *Colletotrichum gloeosporioides* infection revealed by plasma membrane‐enriched proteomics. The heatmap displays DAPs belonging to the category of Receptor and Transporter. The transporters include ABC transporter, Aquaporin, Potassium transporter, GABA transporter, Inositol transporter, Auxin transporter and Oligopeptide transporter. Red arrows: Significant upregulation (fold change > 1.5, *p* < 0.05); Blue arrows: Significant downregulation (fold change < 0.67, *p* < 0.05); ns: Not significant. Value represents the abundance of protein calculated based on the peak intensities of the peptides.

**Figure S7.** The coding sequence (CDS) analysis of *SgATL31*. (a) The CDS sequence alignment of *SgATL31* between RY2 and 2001–84. SgATL31‐1 and SgATL31‐2 represent the CDS of *SgATL31* amplified from RY2 and 2001–84, respectively. SgATL31 represents the sequence from the transcriptome data of RY2. Blue marks the conserved sequences. (b) The phylogenetic tree analysis of ATL31.

**Figure S8.** Molecular validation of *SgATL31*‐overexpressing *Arabidopsis* transgenic lines. Transgenic lines (OE‐1/2) are verified by RT‐PCR. M, DL2000 Plus DNA Marker (Vazyme, MD101‐02); CK, PCR negative control (water template); Col‐0, Wild‐type *Arabidopsis* control; OE‐1/2, Transgenic *Arabidopsis* lines overexpressing *SgATL31*. Upper panel, *SgATL31* ORF amplification (primers, *SgATL31*‐OE‐F/R); Bottom panel, Internal reference gene *QACT* amplification (primers, *QACT*‐qPCR‐F/R).

**Figure S9.** Bimolecular fluorescence complementation (BiFC) analysis of SgATL31 (and its mutant SgATL31^C140H142A^) interactions with AtCPK28, SgCPK28, and SgSYP121 in *N. benthamiana*. The indicated BiFC constructs are co‐expressed into *N. benthamiana*, and fluorescence signals are visualised by confocal microscopy. Interactions between AtATL31 (or its mutant AtATL31^C143H145A^) and AtCPK28 serve as the positive control. Bars, 20 μm.

**Figure S10.** Bimolecular fluorescence complementation (BiFC) analysis of SgATL31 (and its mutant SgATL31^C140H142A^) interactions with AtCPK28 in *Arabidopsis* protoplasts. The indicated BiFC constructs are co‐expressed into *Arabidopsis* protoplasts, and fluorescence signals are visualised by confocal microscopy. Interactions between AtATL31 (or its mutant AtATL31^C143H145A^) and AtCPK28 serve as the positive control. Bars, 2 μm.

**Data S1.** Evaluation of disease resistance in 40 stylo accessions, extraction and enrichment of plasma membrane proteins, proteomic analysis, extraction and transformation of stylo protoplasts.

## Data Availability

All data supporting the current findings are available in the paper and Supporting Information. The raw data of PM proteomics and phosphoproteomics have been deposited to the ProteomeXchange Consortium via the PRIDE (Perez‐Riverol et al. 2022) partner repository with dataset identifiers PXD059433 and PXD059781, respectively. The information of *SgATL31* has been uploaded to NCBI with accession number PQ862150. The complete datasets generated during this study, including phenotypic measurements, enzyme activity assays, and original Western blot images, have been deposited in FigShare (DOI: 10.6084/m9.figshare.28937324).
