# Supplementary figures and images for: E3‐Ubiquitin Ligase SgATL31 Promotes Anthracnose Resistance in Stylosanthes by Modulating ROS Burst and Antioxidant Defence: A Proteomic and Functional Study
Source: Mol Plant Pathol. 2025 Jul 7;26(7):e70122. doi: 10.1111/mpp.70122 (PMC12234378; doi:10.1111/mpp.70122)

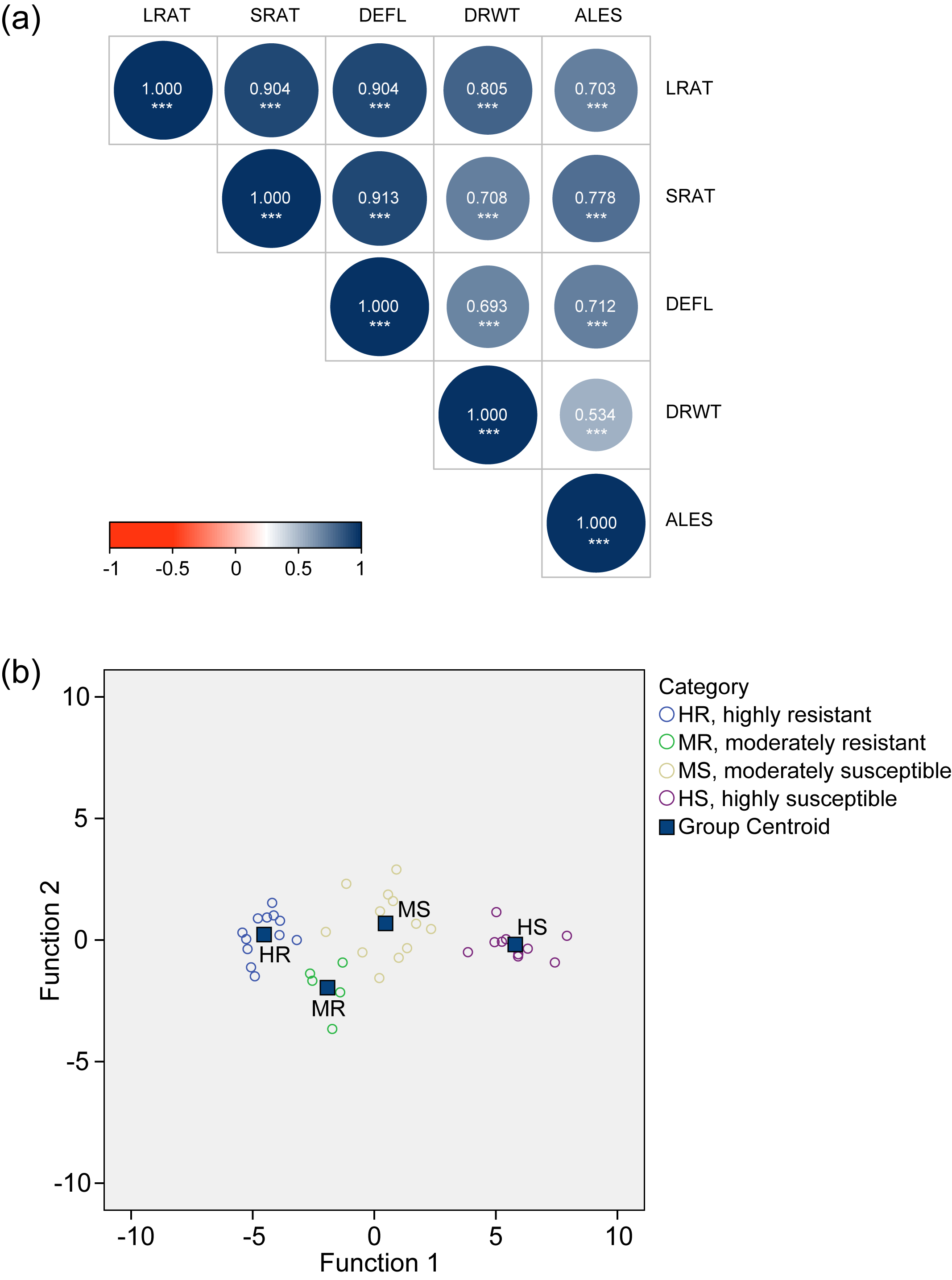

Supplement: Supplementary file 20 — Figure S2. Correlation and discriminant analysis of five disease index (LRAT, SRAT, DEFL, ALES and DRWT). (a) Correlation analysis. Circle sizes represent the magnitude of Pearson’s correlation coefficients. Embedded numerical values show exact correlation coefficients. Colour intensity indicates direction (blue, positive correlations; red, negative correlations). Asterisks mark statistically significant correlations (***p < 0.001). (b) Discriminant analysis. Blue, Highly Resistant (HR) accessions; Green, Moderately Resistant (MR) accessions; Yellow, Moderately Susceptible (MS) accessions; Purple, Highly Susceptible (HS) accessions. [file MPP-26-e70122-s009.tif]

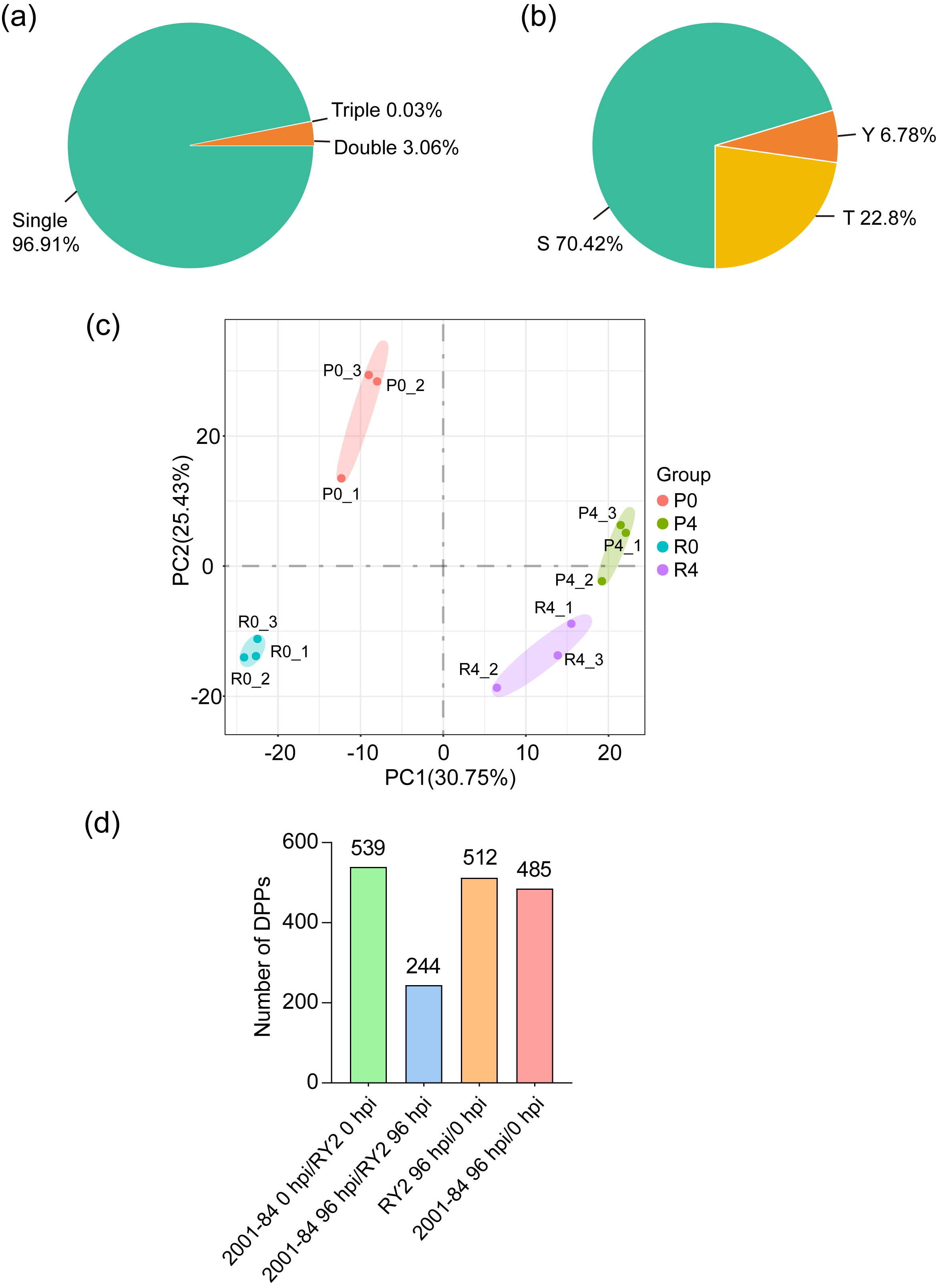

Supplement: Supplementary file 21 — Figure S3. Phosphoproteomic profiling of 2001–84 and RY2 accession in response to Colletotrichum gloeosporioides infection. (a) Phosphosite multiplicity distribution. The percentage of single‐ (one site), double‐ (two sites), and triple‐phosphorylated (three sites) peptide are shown. (b) Phosphoamino acid distribution. The percentage of phosphorylation events on serine (S), threonine (T), and tyrosine (Y) residues are quantified. (c) Principal component analysis (PCA) of phosphorylation profiles. P0 and P4 indicate the samples from RY2 collected at 0 hpi and 96 hpi, respectively. R0 and R4 indicate the samples from 2001–84 collected at 0 hpi and 96 hpi, respectively. Biological triplicates are represented by same‐coloured dots (n = 3 per condition). (d) The number of differentially phosphoproteins (DPPs). Colour codes indicate the number of DPPs in different comparison groups: Green, genotypic differences at 0 hpi (2001–84 vs. RY2); Blue, genotypic differences at 96 hpi (2001–84 vs. RY2); Orange, RY2 temporal changes (96 hpi vs. 0 hpi); Pink, 2001–84 temporal changes (96 hpi vs. 0 hpi). [file MPP-26-e70122-s023.tif]

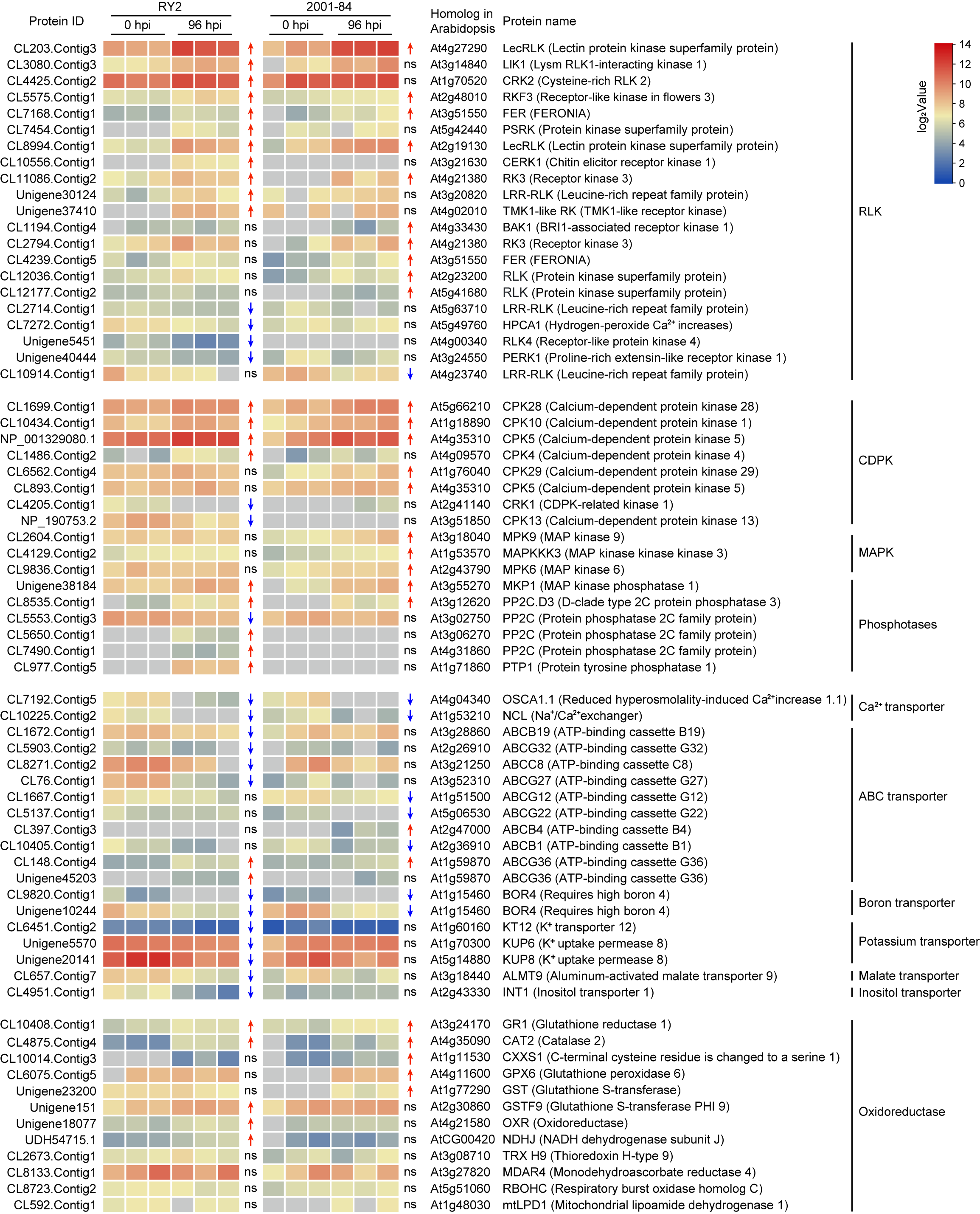

Supplement: Supplementary file 22 — Figure S4. Heatmap analysis of differentially phosphoproteins (DPPs) in RY2 and 2001–84 during Colletotrichum gloeosporioides infection revealed by phosphoproteomics. The heatmap displays DPPs belonging to the category of Protein kinases, Phosphatases, Transporters and Oxidoreductases. The protein kinases include receptor‐like kinase (RLK), calcium‐dependent protein kinase (CDPK), and mitogen‐activated protein kinase (MAPK). The transporters include Ca2+ transporter, ABC transporter, Boron transporter, Potassium transporter, Malate transporter and Inositol transporter. Red arrows: Significant upregulation (fold change > 1.5, p < 0.05); Blue arrows: Significant downregulation (fold change < 0.67, p < 0.05); ns: Not significant. Value represents the abundance of phosphoprotein calculated based on the peak intensities of the peptides. [file MPP-26-e70122-s002.tif]

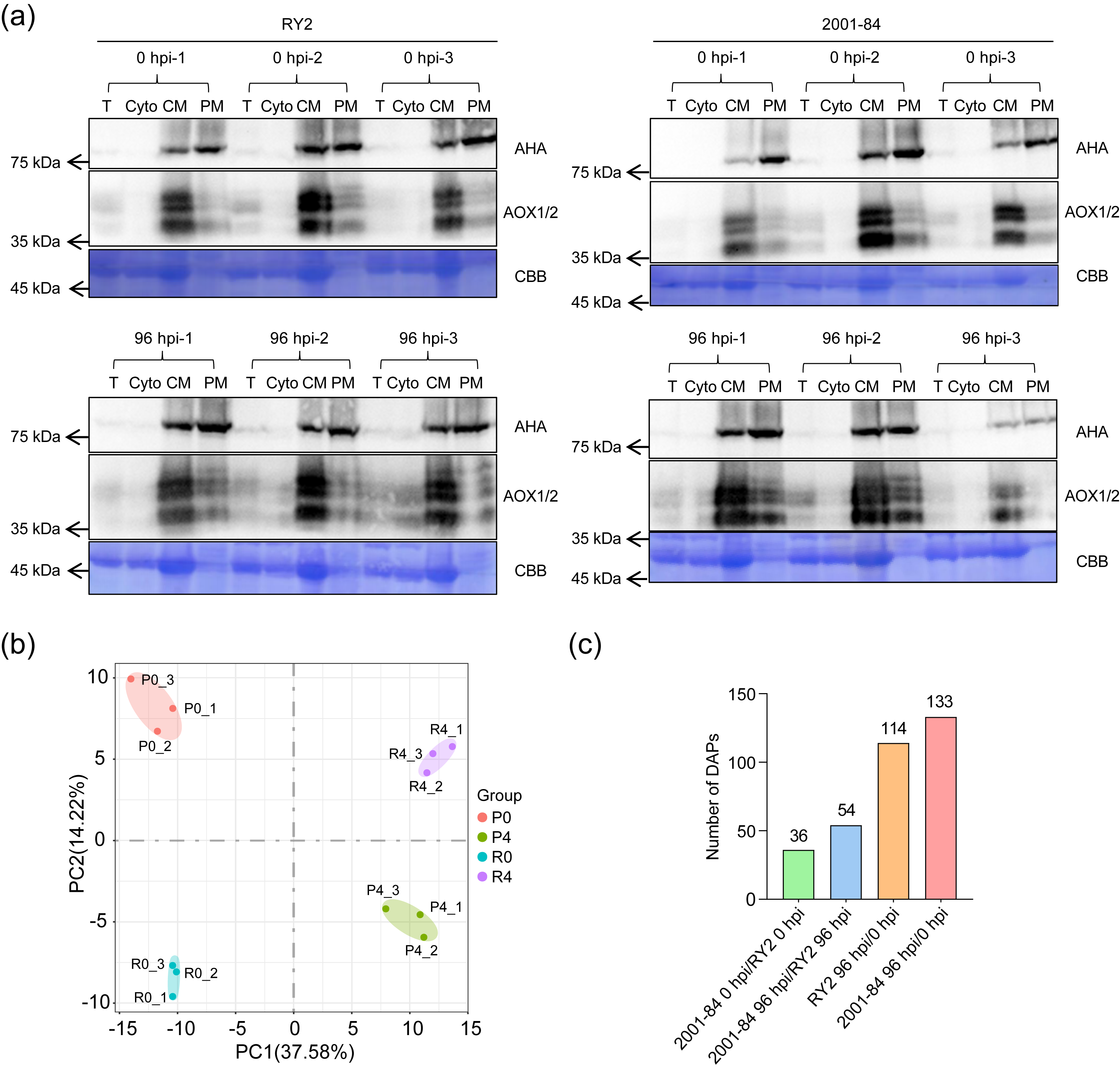

Supplement: Supplementary file 23 — Figure S5. Plasma membrane‐enriched proteomic profiling of 2001–84 and RY2 in response to Colletotrichum gloeosporioides infection. (a) Immunoblot analysis of subcellular fractions using organelle‐specific antibodies. Subcellular fractions: T, total homogenate; Cyto, cytosolic fraction; CM, crude microsomal fraction; PM, plasma membrane‐enriched fraction. Antibodies: AHA (H+‐ATPase, PM marker), AOX1/2 (mitochondrial marker). Total protein loads are detected by coomassie blue staining (CBB). Data represent three biological replicates collected from RY2 and 2001–84 at 0 and 96 h post inoculation (hpi). (b) Principal component analysis (PCA) of PM enriched proteomes. P0 and P4 indicate the samples from RY2 collected at 0 and 96 hpi, respectively. R0 and R4 indicate the samples from 2001–84 collected at 0 and 96 hpi, respectively. Biological triplicates are represented by same‐coloured dots (n = 3 per condition). (c) Differential accumulation of PM proteins. Colour codes indicate the number of differentially accumulated PM proteins (DAPs) in different comparison groups: Green, Genotypic differences at 0 hpi (2001–84 vs. RY2); Blue, Genotypic differences at 96 hpi (2001–84 vs. RY2); Orange, RY2 temporal changes (96 hpi vs. 0 hpi); Pink, 2001–84 temporal changes (96 hpi vs. 0 hpi). [file MPP-26-e70122-s018.tif]

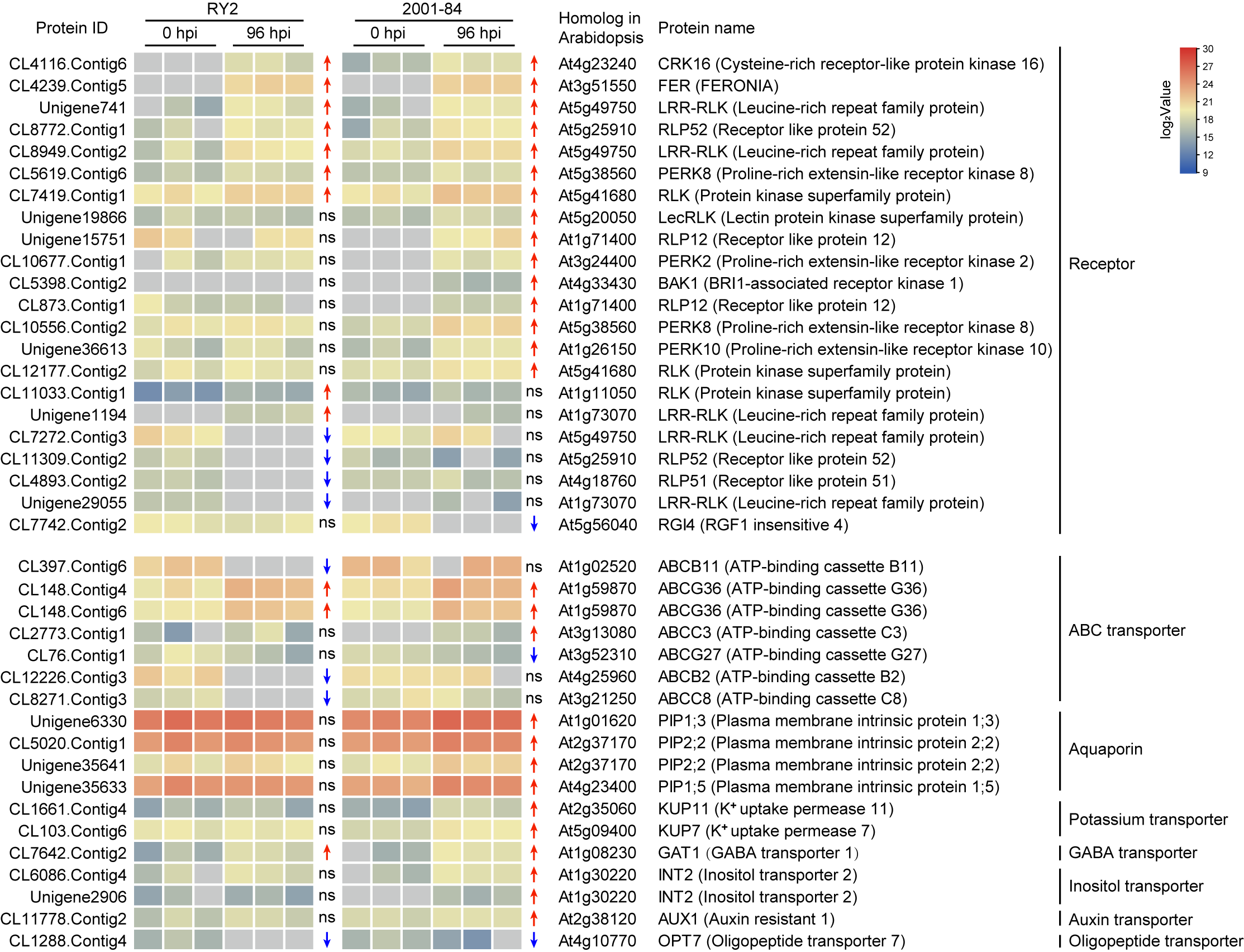

Supplement: Supplementary file 24 — Figure S6. Heatmap analysis of differentially accumulated proteins (DAPs) in RY2 and 2001–84 during Colletotrichum gloeosporioides infection revealed by plasma membrane‐enriched proteomics. The heatmap displays DAPs belonging to the category of Receptor and Transporter. The transporters include ABC transporter, Aquaporin, Potassium transporter, GABA transporter, Inositol transporter, Auxin transporter and Oligopeptide transporter. Red arrows: Significant upregulation (fold change > 1.5, p < 0.05); Blue arrows: Significant downregulation (fold change < 0.67, p < 0.05); ns: Not significant. Value represents the abundance of protein calculated based on the peak intensities of the peptides. [file MPP-26-e70122-s015.tif]

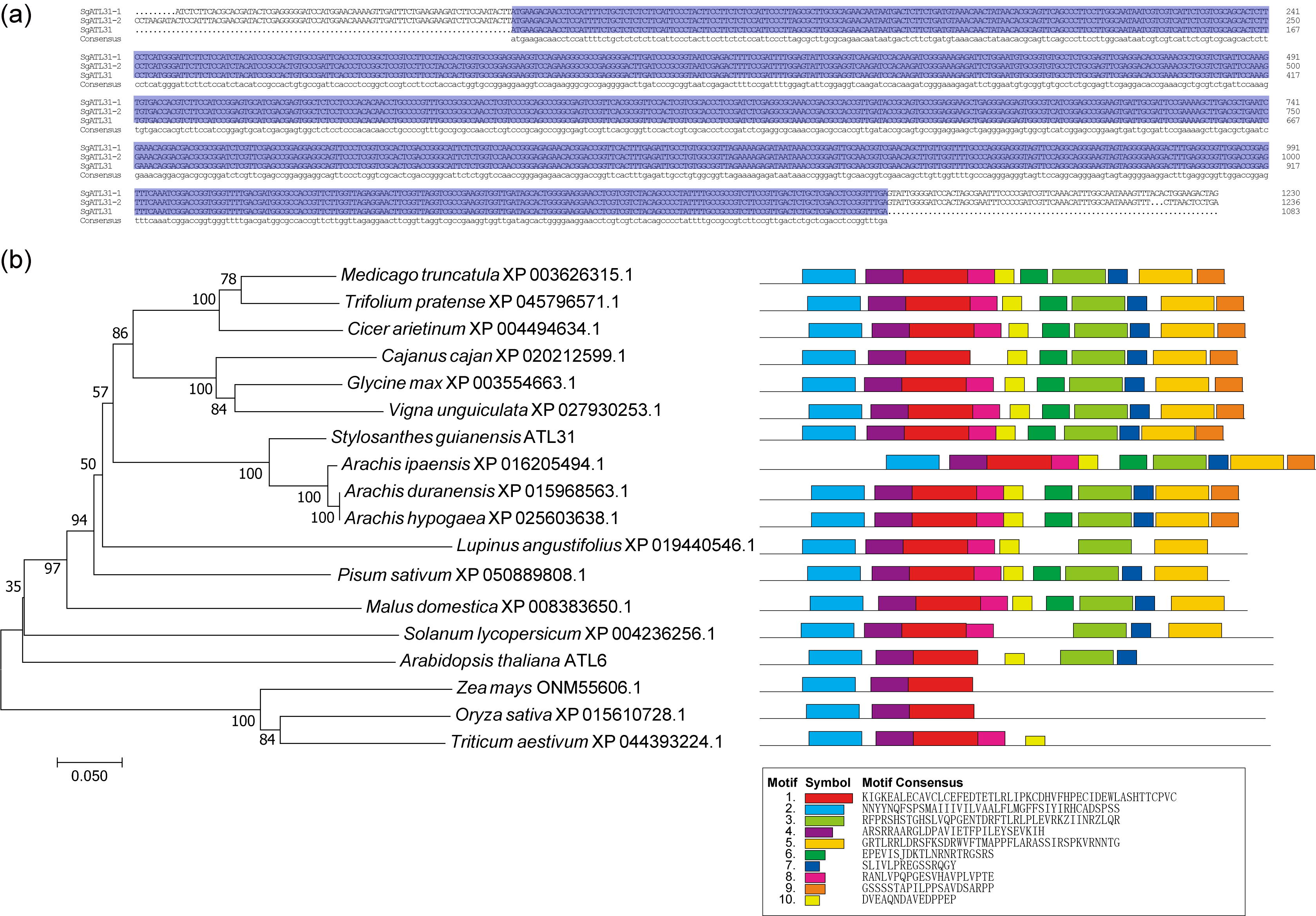

Supplement: Supplementary file 25 — Figure S7. The coding sequence (CDS) analysis of SgATL31. (a) The CDS sequence alignment of SgATL31 between RY2 and 2001–84. SgATL31‐1 and SgATL31‐2 represent the CDS of SgATL31 amplified from RY2 and 2001–84, respectively. SgATL31 represents the sequence from the transcriptome data of RY2. Blue marks the conserved sequences. (b) The phylogenetic tree analysis of ATL31. [file MPP-26-e70122-s004.tif]

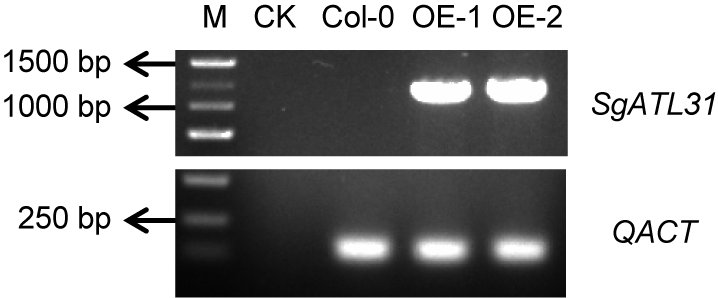

Supplement: Supplementary file 26 — Figure S8. Molecular validation of SgATL31‐overexpressing Arabidopsis transgenic lines. Transgenic lines (OE‐1/2) are verified by RT‐PCR. M, DL2000 Plus DNA Marker (Vazyme, MD101‐02); CK, PCR negative control (water template); Col‐0, Wild‐type Arabidopsis control; OE‐1/2, Transgenic Arabidopsis lines overexpressing SgATL31. Upper panel, SgATL31 ORF amplification (primers, SgATL31‐OE‐F/R); Bottom panel, Internal reference gene QACT amplification (primers, QACT‐qPCR‐F/R). [file MPP-26-e70122-s021.tif]

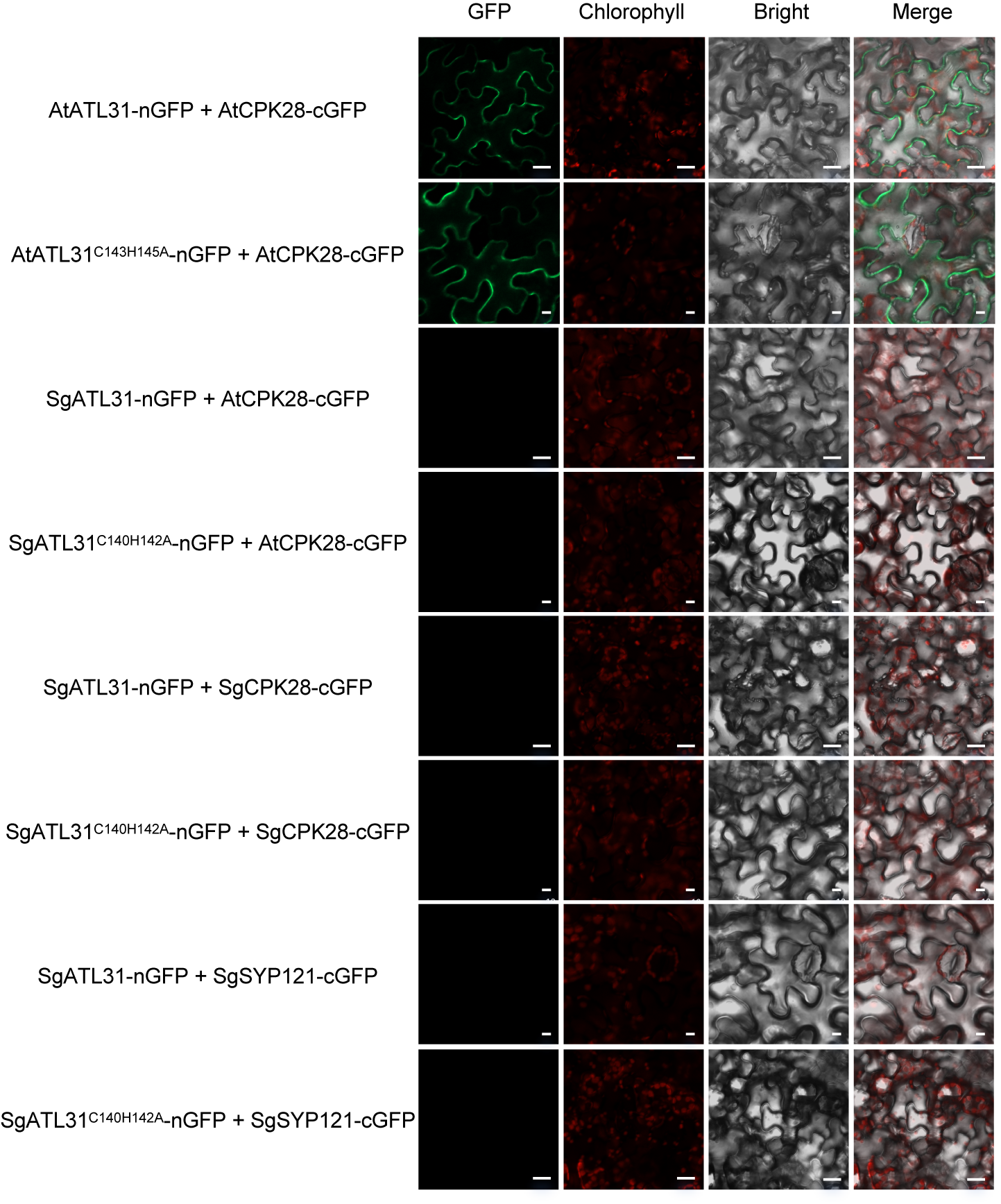

Supplement: Supplementary file 27 — Figure S9. Bimolecular fluorescence complementation (BiFC) analysis of SgATL31 (and its mutant SgATL31C140H142A) interactions with AtCPK28, SgCPK28, and SgSYP121 in N. benthamiana. The indicated BiFC constructs are co‐expressed into N. benthamiana, and fluorescence signals are visualised by confocal microscopy. Interactions between AtATL31 (or its mutant AtATL31C143H145A) and AtCPK28 serve as the positive control. Bars, 20 μm. [file MPP-26-e70122-s007.tif]

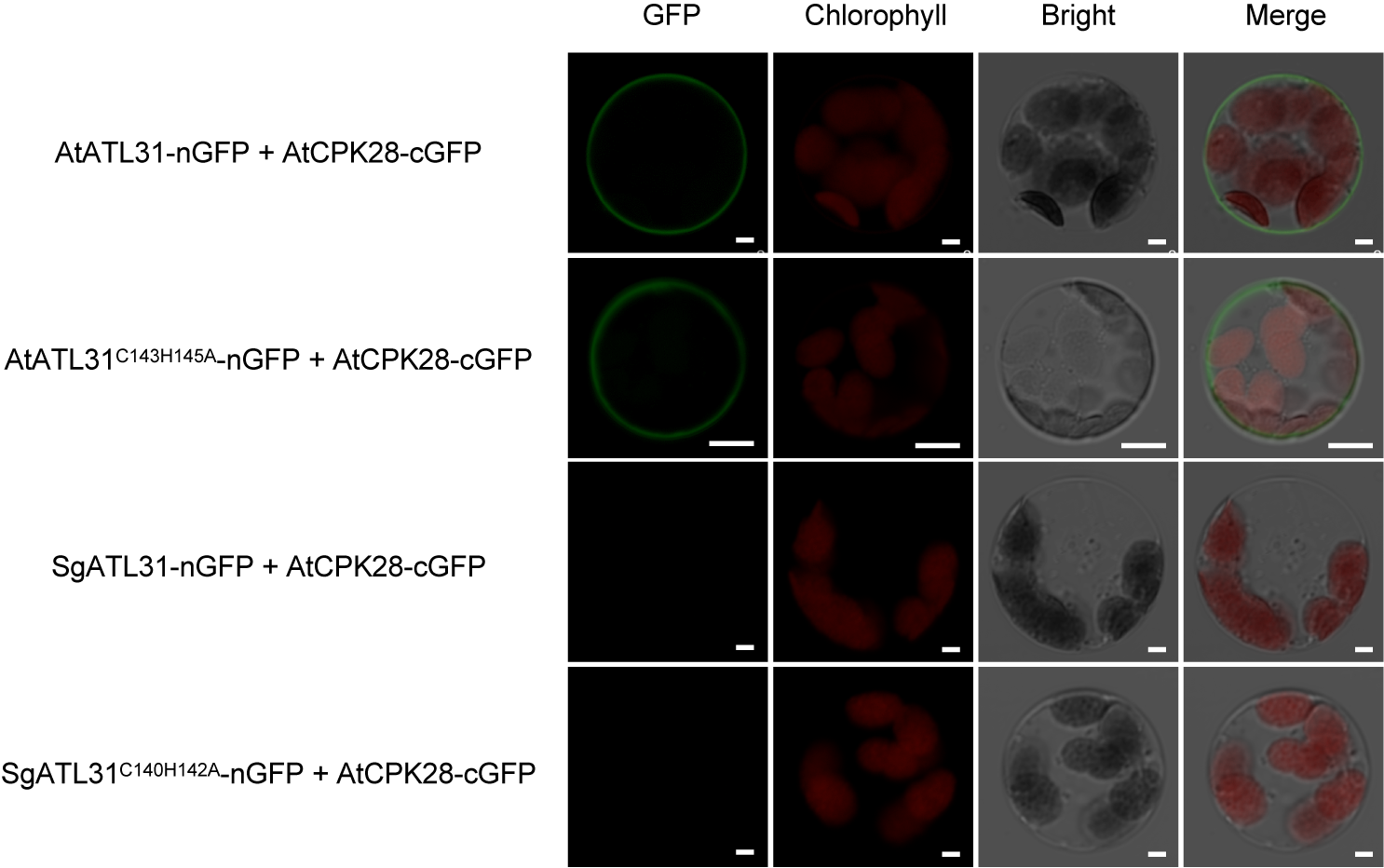

Supplement: Supplementary file 28 — Figure S10. Bimolecular fluorescence complementation (BiFC) analysis of SgATL31 (and its mutant SgATL31C140H142A) interactions with AtCPK28 in Arabidopsis protoplasts. The indicated BiFC constructs are co‐expressed into Arabidopsis protoplasts, and fluorescence signals are visualised by confocal microscopy. Interactions between AtATL31 (or its mutant AtATL31C143H145A) and AtCPK28 serve as the positive control. Bars, 2 μm. [file MPP-26-e70122-s003.tif]
