# Supplementary material for: E3‐Ubiquitin Ligase SgATL31 Promotes Anthracnose Resistance in Stylosanthes by Modulating ROS Burst and Antioxidant Defence: A Proteomic and Functional Study
Source: Mol Plant Pathol. 2025 Jul 7;26(7):e70122. doi: 10.1111/mpp.70122 (PMC12234378; doi:10.1111/mpp.70122)
Supplement: Supplementary file 29 — Data S1. Evaluation of disease resistance in 40 stylo accessions, extraction and enrichment of plasma membrane proteins, proteomic analysis, extraction and transformation of stylo protoplasts. [file MPP-26-e70122-s027.docx]

**Supplementary methods**

1.1 Evaluation of stylo germplasm for the resistance to *C. gloeosporioides*

The resistance index that Leaf disease severity (LRAT), stem disease severity (SRAT), percentage defoliation (DEFL) were analyzed at 5 days after inoculation, and Area of lesions/leaf (ALES) and Dry weight index (DRWT) were analyzed at 7 days after inoculation. LRAT was assessed separately using a six-level scale where 0 = no visible disease, 1 = 0 - 12.5% tissue necrotic, 2 = 12.5 - 25% tissue necrotic, 3 = 25 - 50% tissue necrotic, 4 = 50 - 75% tissue necrotic, 5 = 75 - 100% tissue necrotic. SRAT was assessed using a six-level scale where 0 = no visible disease, 1 = 0 - 1% tissue necrotic, 2 = 1 - 10% tissue necrotic, 3 = 10 - 25% tissue necrotic, 4 = 25 - 50% tissue necrotic, 5 = 50 - 100% tissue necrotic. DEFL was counted on the ratio of the number of fallen leaves to the total number of leaves after inoculation. ALES represented the percentage of lesion area of the first, second, and third new leaves. DRWT indicated the percentage of dry weight loss. Then, the result of DEFL, ALES and DRWT were converted to six-level according to the disease grade of LRAT. LRAT, SRAT, DEFL and ALES of 10 plants, and DRWT of 7 plants were assessed for each germplasm, and the experiments were repeated 3 times. For comprehensively analysis, the resistance index of LRAT, SRAT, DEFL, ALES and DRWT of 40 stylos was calculated as described by Wan et al. (2022). The ward’s cluster analysis of five resistance index and discriminant analysis of the clustering results were performed using IBM SPSS Statistics 20 software, and the interval was set as the square Euclidean distance (Solberg, 1978).

1.2 Extraction and enrichment of plasma membrane proteins

Extraction and enrichment of plasma membrane proteins were performed as described previously (Yang et al., 2022). Briefly, the samples were ground under liquid nitrogen, and dissolved with 10 mL Buffer H per 2 g sample. The homogenate was centrifuged (10,000 r/min) at 4°C for 10 min, then the supernatant was transferred to the ultracentrifuge tube, and was centrifuged (45,000 r/min) at 4°C for 30 min to pellet the crude microsomal fractions. The pellets were suspended and incubated in 2 mL of buffer H with 0.02% w/v Brij-58 (SigmaAldrich, America) per mg of microsomal protein on ice for 30 min, and subsequently ultracentrifuged (45,000 r/min) at 4°C for 30 min. The Brij-58 treatment and ultracentrifugation were repeated one more time to obtain the final enriched plasma membrane protein fraction.

1.3 Western blot

The western blot was performed as previously described (Yang et al., 2022). The isolated total proteins or PM enriched fraction were re-dissolved in 5× sample buffer, then separated by 10% SDS-PAGE and transferred to PVDF membrane at 60 V for 2 h, then sealed with 2% milk for 1 h, subsequently incubated with anti-AHA (H^+^-ATPase, plasma membrane marker, Agrisera) and anti-AOX1/2 (alternative oxidase isoforms 1/2, mitochondrial inner membrane marker, Agrisera) at 4°C overnight. Horseradish peroxidase-linked goat anti-rabbit antibody (Cell signaling and technologies, America) were used to incubate PVDF membrane, then the protein abundance was detected by chemiluminescence. The PVDF membrane was stained with coomassie brilliant blue R-250 to visualize levels of total protein.

1.4 Proteomic analysis

The samples for phosphoproteomic analysis were sent to BGI Tech Solutions Co., Ltd. (BGI-Tech, Shenzhen, China). The proteins were extracted and enzymatically hydrolyzed at 37°C for 4 hours, then desalted by the Strata X column. Phosphopeptides were enriched by TiO_2_, and the high pH reverse phase separation technique (HpHRP) was used to separate peptides. Subsequently, an equal amount of peptides from each sample were mixed into the WATERS nanl/upLC liquid phase system for liquid phase separation. Then samples were performed with dependent acquisition (DDA) and data independent acquisition (DIA) detection by Fusion Lumos (Thermo Fisher Scientific, San Jose, CA).

The samples for PM enriched proteomic analysis were first enriched for PM following the method described in section 1.2, and the effective enrichment was confirmed by western blot analysis described in section 1.3. Then, the PM enriched samples were sent to BGI Tech Solutions Co., Ltd. for LC-MS/MS analysis and protein identification. The samples were enzymatically hydrolyzed at 37°C for 4 hours, then desalted by the Strata X column. The high pH reverse phase separation technique (HpHRP) was used to separate peptides. Subsequently, an equal amount of peptides from each sample were mixed into the LC-20AD liquid phase system for liquid phase separation. Then samples were performed with dependent acquisition (DDA) and data independent acquisition (DIA) detection by timsTOF Pro.

1.5 Extraction and transformation of stylo protoplast

Protoplast extraction and transformation were performed as described by previously method (Gao et al., 2023). Briefly, protoplasts were extracted from stylo mesophyll cell by 1.5% Cellulase R-10 and 1.25% Macerozyme R-10. Then, the protoplast concentration were adjusted to 6.0×10^5^ /mL using W5 solution (154 mM NaCl + 125 mM CaCl_2_ + 5 mM KCl + 2 mM MES, pH 5.7) and 10 µg plasmids were transformed into the protoplasts.

1.6 Bimolecular fluorescence complementation (BIFC) assay in *Arabidopsis* protoplasts and *N. benthamiana*

The CDS of AtATL31 and AtCPK28 were amplified from Arabidopsis cDNA, while SgATL31, SgCPK28, and SgSYP121 were amplified from 2001-84 cDNA. Using Nimble Cloning, we constructed the following BIFC vectors: AtATL31-nGFP and SgATL31-nGFP (wild-type); AtATL31^C143H145A^-nGFP and SgATL31^C140H142A^-nGFP (RING mutants, generated via site-directed mutagenesis using the Mut Express MultiS Fast Mutagenesis Kit V2, C215-01); AtCPK28-cGFP, SgCPK28-cGFP, and SgSYP121-cGFP. Recombinant vectors were transiently expressed in both *Arabidopsis* protoplasts (fluorescence observed after 24 h) and *N. benthamiana* leaves via GV3101 (psoup-p19) infiltration (fluorescence observed after 72 h). Confocal imaging (Leica TCS SP8) used 488 nm excitation for GFP and 630-700 nm detection for chlorophyll autofluorescence.

Reference

Gao J., L. Y. Yang, S. Z. Zhang, and L. Y. Jiang. 2023. “Establishment of isolation and transient expression system of mesophyll protoplast *Stylosanthes guianensis*.” *Acta Agrestia Sinica* 31: 893-902.

Solberg H. E. 1978. “Discriminant analysis.” *CRC Critical Reviews in Clinical Laboratory Sciences* 9: 209-242.

Wan, M. T., L. Y. Yang, S. Z. Zhang, J. Gao, L. Y. Jiang, and L. J. Luo. 2022. “Real-time PCR for detection and quantification of *C. gloeosporioides* s.l. growth in *Stylosanthes* and *Arabidopsis*.” *Crop Protection* 159: 106021.

Yang, L. Y., J. Gao, M. Z. Gao, L. Y. Jiang, and L. J. Luo. 2022. “Characterization of plasma membrane proteins in *stylosanthes* leaves and roots using simplified enrichment method with a nonionic detergent.” *Frontiers in Plant Science* 13: 1071225.
